# Comparisons of the effects of different flaxseed products consumption on lipid profiles, inflammatory cytokines and anthropometric indices in patients with dyslipidemia related diseases: systematic review and a dose–response meta-analysis of randomized controlled trials

**DOI:** 10.1186/s12986-021-00619-3

**Published:** 2021-10-11

**Authors:** Chao Yang, Hui Xia, Min Wan, Yifei Lu, Dengfeng Xu, Xian Yang, Ligang Yang, Guiju Sun

**Affiliations:** 1 Key Laboratory of Environmental Medicine and Engineering of Ministry of Education, School of Public Health, Southeast University, No. 87, Dingjiaqiao, Gulou District, Nanjing, 210009 People’s Republic of China; 2 Department of Nutrition and Food Hygiene, School of Public Health, Southeast University, Nanjing, 210009 People’s Republic of China

**Keywords:** Flaxseed, Lipid profiles, Inflammatory cytokines, Anthropometric indices, Randomized clinical trials, Dyslipidemia related diseases, Meta-analysis, Dose–response

## Abstract

**Background:**

Flaxseed is widely used as a functional food for its rich sources of linolenic acid, lignans and dietary fibers in the world. This systematic review and dose–response meta-analysis on randomized controlled trials (RCTs) is first to evaluate effects of different flaxseed products (whole flaxseed, oil and lignans) on lipid profiles, inflammatory and anthropometric parameters in patients with dyslipidemia related diseases.

**Methods:**

Literature search was performed in PubMed, Embase, Cochrane Central, Scopus, and Web of Science from the inception dates to January, 2020. Weighted mean differences with the 95% confidence interval (CI) were pooled using fix or random-effects models.

**Results:**

Thirty-one RCTs involving 1,698 participants were included. The present meta-analysis revealed that flaxseed consumption had an overall beneficial effect on serum TC, LDL-C, TG, apo B and IL-6 in patients with dyslipidemia related diseases, but not on apo A, HDL-C, hs-CRP, CRP and anthropometric indices. However, different flaxseed products showed obviously different effects. Whole flaxseed supplementation significantly reduced TC (− 11.85 mg/dl, 95% CI − 20.12 to − 3.57, *P* = 0.005), LDL-C (− 10.51 mg/dl, 95% CI − 14.96 to − 6.06, *P* < 0.001), TG (− 19.77 mg/dl, 95% CI − 33.61 to − 5.94, *P* = 0.005), apolipoprotein B (− 5.73 mg/dl, 95% CI − 7.53 to − 3.93, *P* < 0.001), TC/HDL-C (− 0.10, 95% CI − 0.19 to − 0.003, *P* = 0.044) and weight (− 0.40 kg, 95% CI − 0.76 to − 0.05, *P* = 0.027); Lignans supplementation significantly reduced TC (− 17.86 mg/dl, *P* = 0.004), LDL-C (− 15.47 mg/dl, *P* < 0.001) and TC/HDL-C (− 0.45, *P* = 0.04). Although flaxseed oil supplementation had no such lowering-effect on lipid, meta-analysis revealed its lowering-effect on IL-6 (− 0.35 pg/ml, *P* = 0.033) and hs-CRP (− 1.54 mg/l, *P* = 0.004). Subgroup analysis revealed that whole flaxseed decreased TC, LDL-C and TG levels irrespective of country and the intervention time prescribed, but was more pronounced when the dose of whole flaxseed was ≤ 30 g/day (TC: WMD − 13.61 mg/mL; LDL-C: WMD − 10.52 mg/mL; TG: WMD − 23.52 mg/mL), rather not a dose > 30 g/day. Moreover, a linear relationship between dose of whole flaxseed and absolute changes in C-reactive protein (*P* = 0.036) and a nonlinear relationship between with IL-6 (*P* < 0.001) were detected.

**Conclusions:**

Flaxseed intervention suggested the positive effects on lipid profiles, inflammatory cytokines and anthropometric indices in patients with dyslipidemia related diseases. Of these, whole flaxseed and lignans play an important role in reducing blood lipid, while flaxseed oil mainly plays in anti-inflammatory. Lipid- and weight-lowering was significant when whole flaxseed was consumed at doses < 30 mg/d, for lipid status with mixed dyslipidemia and patients with BMI > 25.

**Graphic abstract:**

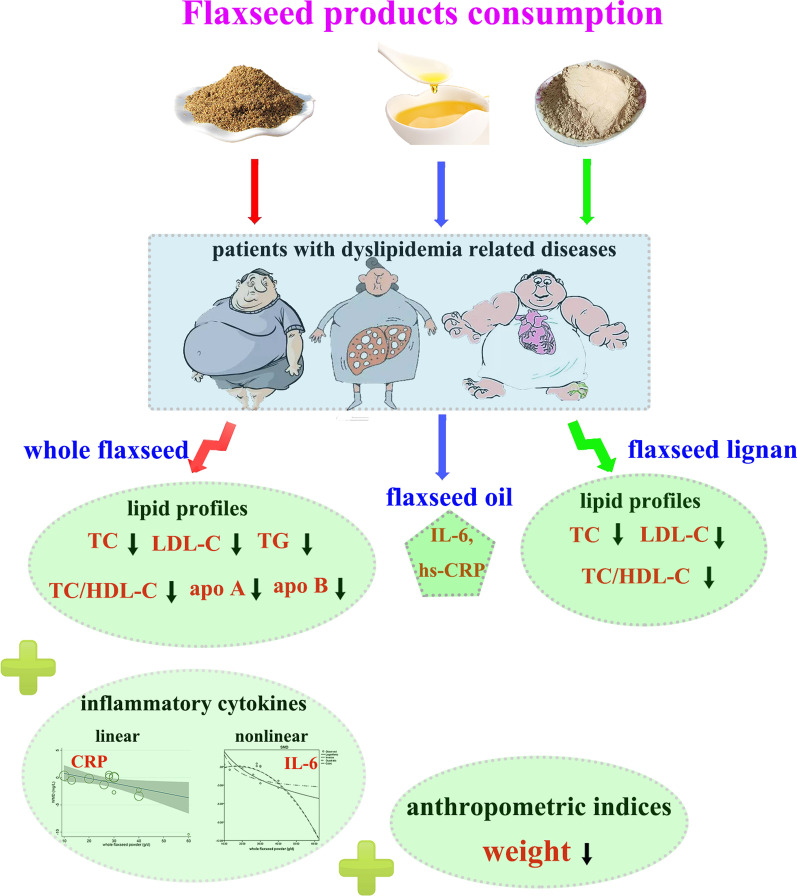

**Supplementary Information:**

The online version contains supplementary material available at 10.1186/s12986-021-00619-3.

## Introduction

Flaxseed (*Linum usitatissimum* L.) is widely used as a functional food in the world, because it is a rich source of linolenic acid, lignans (secoisolariciresinol diglucoside [SDG]), and soluble and both insoluble dietary fibers, which are related to cholesterol control [1, 2]. Flaxseed lignans plays a role in antiatherosclerosis including anti-inflammatory, antioxidant, potent angiogenic, and antiapoptotic properties [3, 4]. Flaxseeds are also the best source of α-linolenic acid (ALA), which is associated with human health benefits [5]. The flaxseed fiber also plays roles on reducing the blood glucose and cholesterol levels by delaying and reducing their absorption from the intestine [6, 7].

It has been observed that many lipid/lipoprotein abnormalities are prevalent in obesity and heart problems, such as atherosclerosis and coronary heart disease (CHD) [8], collectively termed as dyslipidemia; these dyslipidemias are often hyperlipidemia which is characterized by elevated serum total cholesterol (TC), triglyceride (TG), low-density lipoprotein cholesterol (LDL-C) and very LDL-C (VLDL-C), and decreased high-density lipoprotein cholesterol (HDL-C) levels. Hyperlipidemia has become one of the greatest risk factors contributing to CHD, and also is associated with lipid disorders, which are considered the cause of atherosclerotic cardiovascular disease [9]. Moreover, nonalcoholic fatty liver disease (NAFLD) also was regarded associated with dyslipidemia [10, 11]. Abnormalities in the lipid profile, specifically hypertriglyceridemia and low levels of HDL-C have been shown to be a strong predisposing issue to dyslipidemia related diseases. Likely, chronic inflammation was represented anther important factor in the development of dyslipidemia related diseases, such as dyslipidemia, atherosclerosis, CVD, metabolic syndrome (MetSyn) and obesity [12].

In recent years, the health effect of flaxseed intervention has aroused great concern in China. There was also a meta-analysis to evaluate the flaxseed effects on blood lipid [13], and several studies evaluated the reducing-inflammation effect regardless of supplement dosage [14, 15]. But few meta-analyses considered the health background of participants i.e., health or be with chronic disease, and almost of them ignored the effect on weight loss of flaxseed intervention. Moreover, to our knowledge, all types of flaxseed were included i.e., oil, lignin and whole flaxseed may exist different health effects. Flax varieties may differ in their unsaturated fatty acid profile together with phenolic lignan composition that can affect the biomedical activity of flaxseed oil. In fact, there is no meta-analysis to distinguish the function of different types of flaxseed products. In light of above, it is necessary to conduct a dose-responsive model of evaluating different flaxseed products consumption and its beneficial healthy effects.

Therefore, we conducted an updated systematic review and dose–response meta-analysis of randomized controlled trials (1) to summarize the pooled effects of flaxseed intervention on patients with dyslipidemia related diseases to detect the most immediate effect not only on lipid profiles, inflammatory cytokines but also anthropometric indices, (2) to distinguish the functions of different types of flaxseed product i.e., oil, lignan and whole flaxseed, and (3) to explore the dose–response relationship between whole flaxseed and flaxseed oil with the above indexes.

## Method

This systematic review was performed according to the Preferred Reporting Items for Systematic Reviews and Meta-analysis (PRISMA) statement [16]. Table 1 shows the population intervention, comparator, outcome, and setting criteria used to perform the systematic review. According to the local legislation, ethical approval is not necessary for this meta-analysis and systematic review.

**Table 1:** PICOS (population, intervention, comparator, outcome, and setting) criteria used to perform the systematic review and meta-analyses

PICOS	Criteria
Population	Patients with dyslipidemia related diseases
Intervention	Flaxseed products consumption (whole flaxseed, flaxseed oil and lignan)
Comparator	Placebo group/control group
Outcome	Lipid profiles (TC, LDL-C, HDL-C, TG and apo B), inflammatory cytokines (IL-6, TNF-α, CRP and hs-CRP) and anthropometric indices (WC, WHR, Weight and BMI)
Setting	a randomized clinical trial; a randomized crossover study

TC, total cholesterol; TG, triglyceride; LDL-C, low-density lipoprotein cholesterol; HDL-C, high-density lipoprotein cholesterol; apo A, apolipoprotein A; apo B, apolipoprotein B; BMI, body mass index; WC, waist circumference; WHR waist-height ration

### Search strategy

A literature search was performed in PubMed, Embase, Cochrane Library, Scopus, and Web of Science (WOS) from the inception dates to January 2020. We systematically searched databases by three independent investigators (C.Y., H.X., and M.W.). The computer-based searches included the key words (flaxseed OR linseed OR "flaxseed oil" OR "flaxseed lignan") in combination with ("non-alcoholic fatty liver disease" OR obesity OR overweight OR dyslipidemia OR hyperlipidemia OR "coronary heart disease" OR atherosclerosis). Moreover, the reference or citation lists from the retrieved articles were checked to search for further relevant studies. The search was limited to studies in humans published in English.

### Definition of variables

Dyslipidemia related diseases were regarded as a series of chronic diseases characterized by lipid metabolism disorder and chronic inflammation, mainly included dyslipidemia, overweight, obesity, MetSyn, NAFLD, and atherosclerosis [17]. Hypertriglyceridemia was defined as a triglycerides level ≥ 150 mg/dL (1.70 mmol/l) and hypercholesterolemia was defined as a total cholesterol level ≥ 240 mg/dL (6.22 mmol/l). We defined body mass index (BMI) categories using the following BMI categories: normal (18.5- < 25 kg/m^2^), overweight (25- < 30 kg/m^2^), and obesity (≥ 30 kg/m^2^). Moreover, waist circumference ≥ 90 cm for men or ≥ 80 cm for women was defined as central obesity [18].

### Inclusion criteria and exclusion strategy

Original studies were included if they met the following criteria: (1) diseases reported were that dyslipidemia, overweight, obesity, MetSyn, NAFLD, and atherosclerosis, (2) randomized clinical trials with a parallel or cross-over design, (3) investigation to the effects of flaxseed products on lipid profiles (TC, LDL-C, HDL-C, TG, apo A and apo B), anthropometric indices (weight, BMI and waist circumference) and inflammatory cytokines (IL-6, hs-CRP, CRP and TNF-α), (4) above-mentioned indicators could be extracted from the report, or providing baseline and end-trial indicators concentrations in both flaxseed and control groups, and (5) having supplementation with flaxseed or its derivatives for at least 2 weeks.

Animal trials, cytological study and publications with insufficient data were excluded. Non-clinical studies, uncontrolled trials, and trials with insufficient data on lipid, anthropometric and inflammatory parameters in flaxseed and control groups were excluded from the meta-analysis.

### Data extraction

Data extraction was conducted using a standardized data collection independently by two investigators (C.Y., X.Y.). Inconsistencies were resolved by discussion until a consensus was reached. Study characteristics [including authors, publication year, sample size, type of the intervention, control treatment, study duration, study design, and participant features (sex, age, BMI, blood lipid data and dyslipidemia state)] were extracted. If a study had some comparisons by different intervention time, different product-types or doses of intervention, we regarded these comparisons as multiple studies. There are three studies [19–21] with two dose comparisons, one study [22] with two intervention time comparisons, and one study [23] with two product types.

### Quality assessment

Assessment of study quality was independently performed by two reviewers (Y.F.L. and D.F.X.) using the Cochrane Collaboration tool for assessing risk of bias in RCTs [24]. The Cochrane tool has seven domains: random sequence generation (selection bias), allocation sequence concealment (selection bias), blinding of participants and personnel (performance bias), blinding of outcome assessment (detection bias), incomplete outcome data (attrition bias), selective outcome reporting (reporting bias), and other potential sources of bias. Each item was classified as low, high, or an unclear risk of bias (if there was insufficient information) [25].

### Quantitative data synthesis

The estimate of principal effect was defined as the weighted mean difference (WMD) in lipid concentrations (net change in mg/dL), anthropometric indices (net change in kg, cm, or kg/cm^2^) and inflammatory cytokines (net change in pg/mL or mg/L) between the subjects assigned to consume flaxseed or its derivatives and those assigned to the control regimens. Standard deviations (SDs) of the mean difference were calculated using correlation coefficient methods referenced in the Cochrane Handbook by the following formula: SD = square root [(SD _baseline_)^2^ + (SD _end_)^2^ − (2R × SD _baseline_ × SD _end_)] [24], assuming a correlation coefficient (R) equal to 0.5 [26]. If standard error of the mean (SE) was only reported in included articles, the following formula [SD = SEM × sqrt (n)] was used to estimate the SD, where *n* is the number of subjects. The net changes in lipid concentrations, anthropometric indices and inflammatory cytokines were calculated by subtracting the change value after control-intervention from that calculated after treatment-intervention.

### Statistical analysis

The meta-analysis was performed by using STATA SE (StataCorp LP, College Station, TX, USA). Q test and I^2^ index were used to assess the heterogeneity among the included studies. Substantial heterogeneity was indicated as *P* < 0.05 in the χ^2^ test and an I^2^ > 50%. The fixed-effect models and random-effects models were used to calculate the WMD and 95% confidence interval (CI) for each variable according to the level of heterogeneity. If significant heterogeneity was present, then a random-effect model was used.

Meta-regression was implemented to examine characteristics of the studies that were hypothesized to influence the observed treatment effects. It was performed to assess the association between the overall estimate of effect sizes with potential moderator variables, including type of flaxseed, BMI categories, lipid status, study country, intervention time and study design (parallel or crossover).

Further subgroup analyses were performed to explore impacts of certain characteristics: type of flaxseed (whole flaxseed, flaxseed oil or flaxseed lignans), country (Asian or Westerner), interventional time (≤ 10 weeks or > 10 weeks), dose of flaxseed products (whole flaxseed: ≤ 30 g/d or > 30 g/d; flaxseed oil: ≤ 10 g/d or > 10 g/d), BMI categories (18.5- < 25 kg/m^2^, 25- < 30 kg/m^2^, and ≥ 30 kg/m^2^) and lipid status (hypercholesterolemia, hypertriglyceridemia, mixed dyslipidemia and non-dyslipidemia). Sensitivity analyses were completed to detect the robustness of the statistical results and analyze possible sources of heterogeneity by excluding studies on a one-by-one basis, followed by the exclusion of those with a high risk of bias. Egger’s test was conducted to quantitatively explore the possible publication bias (significant level = 0.05). All tests were two-tailed and *P* < 0.05 indicated statistical significance.

## Results

### Study selection

A total of 1,856 articles were identified from our initial search (311 from PubMed, 108 from Embase, 828 from WOS, 117 from Cochrane, and 491 from Scopus); 511 articles were excluded during an initial review (with title and abstract). We retrieved the full text for the remaining 126 articles based on PICOS criteria, of which 32 were retrieved for a complete evaluation. Finally, 31 citations met the inclusion after excluding one [27] with incomplete data. The review flow diagram (PRISMA flow diagram) is depicted in Additional file 2: Fig. 1.

#### Characteristics of included studies

Table 2 summarizes the baseline characteristics of the included studies and their participants. Of these studies included in the final analysis, all studies provided published data. Overall, 1,698 participants were assigned into these trials randomly, in that 875 individuals were allocated to the flaxseed intervention group and 823 to the control group. The 31 randomized controlled trials (RCTs) were published from 1993 to 2020, and were conducted in China (n = 3) [19, 28, 29], United States (n = 6) [21, 23, 30–33], Canada (n = 3) [34–36], Japan (n = 1) [20], Greece (n = 3) [37–39], Brazil (n = 5) [22, 40–43], Demark (n = 1) [44], Germany (n = 1) [45], India (n = 1) [46], and Iran (n = 7) [47–53]. Four trials were conducted exclusively in men [20, 37–39], 2 trials were conducted in women [31, 44], the remaining 25 RCTs were conducted in both sexes.

**Table 2:** Demographic characteristics and baseline parameters of the 33 studies selected for analysis

References	Country	Completer/enrollment	Patient features ()	Dyslipidemia state	Age [Mean (SD) or range]	Design	Intervention	WF (g/d)	FLO (g/d)	LIG (g/d)	Duration (weeks)
*Patients with NAFLD*											
Rezaei et al. [47]	Iran	57/68	Patients with NAFLD; BMI ≥ 25 (FLO: 30.1 ± 4.1; SFO: 29.6 ± 3.9) kg/m^2^; Gender: Mixed	Marginal hypertriglyceridemia; TG (FLO: 1.9 ± 1.6; SFO: 1.71 ± 0.99) mmol/L	FLO: 45.5 (8.7) SFO: 40.8 (8.7)	RCT^a^	FLO/ SFO	NA	20 ml/d	NA	12 weeks
Yari et al. [48]	Iran	50/52	Patients with NAFLD; BMI: 30.72 ± 3.31 kg/m^2^; Gender: Mixed	Hypertriglyceridemia; TG: 215.26 ± 85.68 mg/dL	45.02 (10.44)	RCT^a^	LC + brown milled flaxseed/ LC	30 g	NA	NA	12 weeks
*Patients with Atherosclerosis*											
Raygan et al. [51]	Iran	55/60	T2DM patients with CHD; BMI: < 25 and ≥ 25 kg/m^2^; Gender: Mixed	Non dyslipidemia	FLO: 64.1 (9.3) Con: 64.6 (9.1)	RCT^a^	FLO /paraffin	NA	1 g*2 (ALA: 0.4 g)	NA	12 weeks
Edel et al. [34]	Canada	84/110	Patient with peripheral artery disease; BMI: Not reported; Gender: Mixed	Non dyslipidemia	Not reported	RCT^a^	Milled flaxseed/ whole wheat	30 g	NA	NA	12 moths
*Patients with MetSyn*											
Akrami et al. [52]	Iran	52/60	Patients with MetSyn; Weight (FLO: 81.17 ± 11.23, SFO: 84.50 ± 14.89) kg; WC (FLO: 99.42 ± 6.95, SFO: 101.85 ± 10.73) cm; Gender: Mixed	Marginal hyperlipidemia; (HDL < 40 mg/dL for men and < 50 mg/dL for women, TG ≥ 150 mg/dL)	FLO: 48.3 (6.9) SFO: 48.8 (6.4)	RCT^a^	FLO/SFO	NA	25 ml/d	NA	7 weeks
Yari et al. [49]	Iran	44/60	Patients with MetSyn; BMI: 30.90 ± 3.39 kg/m^2^; Gender: Mixed	Hypertriglyceridemia; TG: 217.6 ± 86.44 mg/dL	Flax: 45.8 (10.9) Con: 45.2 (10.3)	RCT^a^	Brown milled flaxseed + LC/ LC	30 g	NA	NA	12 weeks
Wu et al. [28]	China	189/189	Patients with MetSyn; BMI (LC: 25.4 ± 2.4, LCF: 25.1 ± 2.3) kg/m^2^; Gender: Mixed	Hyperlipidemia; TC (LC: 6.1 ± 1.7, LCF: 6.0 ± 1.5) mmol/L; LDL-C (LC: 4.3 ± 1.4, LCF: 4.2 ± 1.3) mmol/L; TG (LC: 1.94 [1.45–2.80], 1.89 [1.36–2.77]) mmol/L	25–65	RCT^a^	LC + flaxseed/LC	30 g	NA	NA	12 weeks
*Patients with Dyslipidemia*											
Torkan et al. [50]	Iran	70/70	Patients with hyperlipidemia; BMI (Flax: 27.28 ± 0.61; Con: 27.68 ± 0.43) kg/m^2^; Gender: Mixed	Hyperlipidemia TC (Flax: 226 ± 6.2, Con: 214.8 ± 5.7) mg/dL; TG (Flax: 226.05 ± 18.7, Con: 213 ± 15.6) mg/dL	Flax: 43.4 (1.1) Con: 40.5 (1.4)	RCT^a^	Raw flaxseed powder/NR	30 g	NA	NA	5.7 weeks (40 days)
Cassani et al. [40]	Brazil	27/27	Men with cardiovascular risk factors; WC ≥ 90 cm; BMI (Flax: 32 ± 3, Con: 32.1 ± 2.8) kg/m^2^; Gender: Men	Hyperlipidemia; TC ≥ 200 mg/dL, LDL-C ≥ 130 mg/dL, HDL-C < 40 mg/dL, TG ≥ 150 mg/dL	Flax: 40 (9) Con: 33 (10)	RCT^a^	Brown flaxseed powder/rice raw powder	60 g	NA	NA	6 weeks
Dittrich et al. [45]	Germany	49/59	Patients with moderate hypertriglyceridemia; BMI (SFO: 28.35 ± 4.25, FLO: 28.10 ± 3.84) kg/m^2^; Gender: Mixed	Moderate hypertriglyceridemia; TG > 1.5 mmol/L	56 (12)	RCT^2^	FLO/SFO	NA	10 g/d (ALA: 7 g)	NA	10 weeks
Saxena et al. [46]	India	50/50	Patients with dyslipidemia; BMI (Flax: 28.48 ± 2.91, Con: 28.90 ± 4.21) kg/m^2^; Gender: Mixed	Hyperlipidemia; TG > 150 mg/dL; TC > 200 mg/dL; LDL-C > 130 mg/dL	40–60	RCT^a^	Roasted flaxseed powder /NR	30 g	NA	NA	3 moths
Soltani et al. [53]	Iran	30/38	Hemodialysis patients with dyslipidemia; BMI (Flax: 25.5 ± 2.0, Con: 27.0 ± 1.0) kg/m^2^; Gender: Mixed	Hyperlipidemia; TG > 200 mg/dL and/or HDL-C < 40 mg/dL	Flax: 54.0 (4.0) Con: 54.5 (4.0)	RCT^a^	Ground flaxseed/usual diet	40 g	NA	NA	8 weeks
Gillingham et al. [35]	Canada	36/39	Subjects with hypercholesterolemia; BMI: 28.56 ± 4.62 kg/m^2^; Gender: Mixed	Hypercholesterolemia; LDL-C > 3.0 mmol/L	47.49 (11.93)	RCT^2^	HOCO + FLO/HOCO	NA	NA	NA	4 weeks
Fukumitsu et al. [20]	Japan	16/20	Men with moderately hypercholesterolemic; BMI (LIG: 23.5 ± 1.0, 23.8 ± 1.2; Con: 25.5 ± 1.7) kg/m^2^; Gender: Men	Hypercholesterolemia 4.65 ≤ TC ≤ 6.21 mmol/L (180–240 mg/mL)	21–57	RCT^a^	LIG/placebo (corn starch)	NA	NA	0.02	12 weeks
		16/20						NA	NA	0.1	
Zhang et al. [19]	China	35/44	Men and women with hypercholesterolemia; BMI: 26–27 kg/m^2^; Gender: Mixed	Hypercholesterolemia; LDL-C ≥ 3.62 mmol/L	53.5–58.3	RCT^a^	LIG/placebo (maltodextrin)	NA	NA	0.3	8 weeks
		37/44						NA	NA	0.6	
Bloedon et al. [23]	US	50/62	Men and post-menopausal women with hypercholesterolemia; BMI: (Flax: 27.4 ± 4.4, Con: 28.1 ± 5.1) kg/m^2^; Gender: Mixed	Hypercholesterolemia; LDL-C of 130–200 mg/dL, TG < 600 mg/dL	44–75	RCT^a^	Flaxseed/wheat bran	20 g	NA	NA	5 weeks
											10 weeks
Patade et al. [30]	US	26/37	Postmenopausal women with hypercholesterolemia (moderate); BMI: Not reported. Gender: Woman	Hypercholesterolemia; TC: ≥ 5.1 to ≤ 9.8 mmol/L	47–63	RCT^a^	Flaxseed/muffins and white bread	30 g	NA	NA	3 moths (12.9 weeks)
Paschos et al. [37]	Greece	87/87	Men with dyslipidemia; BMI (FLO: 28 ± 3, SAO: 28 ± 4) kg/m^2^; Gender: Men	Hyperlipidemia; TC > 200 mg/dL, and/or HDL-C < 40 mg/dL	SAO: 52.0 (1.0) FLO: 54.1 (1.6)	RCT^a^	FLO/SAO	NA	15 ml/d (ALA: 8 g)	NA	12 weeks
Rallidis et al. [39]	Greece	76/90	Men with dyslipidemia; BMI: (ALA: 28.42 ± 3.44, 28 ± 3.19) kg/m^2^; Gender: Men	Hyperlipidemia; TC (ALA: 232 ± 34, LA: 234 ± 46) mg/dL; LDL-C (ALA: 154 ± 37, LA: 154 ± 43) mg/dL; TG (ALA: 155 ± 84, LA: 173 ± 113) mg/dL	ALA: 50.4 (7.3) LA: 52.0 (7.7)	RCT^a^	Linseed oil/LA	NA	15 ml/d (ALA: 8.1 g)	NA	12 weeks
Rallidis et al. [38]	Greece	76/90	Men with dyslipidemia; BMI: (ALA: 28.42 ± 3.44, 28 ± 3.19) kg/m^2^; Gender: Men	Hyperlipidemia; TC (ALA: 232 ± 34, LA: 234 ± 46) mg/dL; LDL-C (ALA: 154 ± 37, LA: 154 ± 43 mg/ dL); TG (ALA: 155 ± 84, LA: 173 ± 113 mg/ dL)	ALA: 50.4 (7.3) LA: 52.0 (7.7)	RCT^a^	Linseed oil//SAO	NA	15 ml/d (ALA: 8 g)	NA	12 weeks
Jenkins et al. [36]	Canada	29/37	Men and postmenopausal women with hyperlipidemia; BMI (24.9 ± 0.5 [mean ± SEM]; range: 19.6–29.8) kg/m^2^; Gender: Mixed	Hyperlipidemia; [LDL-C > 4.1 mmol/L (160 mg/dL) or TG > 2.3 mmol/L (200 mg/dL)]	57 (2)	RCT^a^	Defatted flaxseed/wheat bran	50 g	NA	NA	3 weeks
Arjmandi et al. [31]	US	34/38	Postmenopausal women with hypercholesterolemia; Gender: Woman	mild, moderate, or severely hypercholesterolemia; (5.85–9.05) mmol/L	56.3 (6.5)	RCT^a^	WF/ sunflower seed	68 g	NA	NA	6 weeks
Bierenbaum et al. [32]	US	15/15	Subjects with hypercholesterolemia; None were excessively overweight (mean 154 ± 4.8 lbs); Gender: Mixed	Hypercholesterolemia TC > 240 mg/dL	52.2 (1.8)	RCT^2^	VE + Flaxseed powder/VE	15 g	NA	NA	12 weeks
*Subjects with overweight or obese*											
Yang et al. [29]	China	66/73	Hypertensive comorbid with abdominal obesity; BMI (FLO: 26.83 ± 3.06, CO: 26.78 ± 2.98) kg/m^2^; WC (97.42 in men, 95.74 in women) cm; Gender: Mixed	Non dyslipidemia	FLO: 56.6 (7.8) CO: 58.4 (6.87)	RCT^a^	FLO/ corn oil	NA	4 g/d (ALA: 2.5 g)	NA	13 weeks/90 days
de Oliveira et al. [41]	Brazil	76/79	Obese or overweight non-Diabetic elderly patients; BMI ≥ 27 (FLO: 32.7 ± 4.62, OO: 34.4 ± 3.97) kg/m^2^; Gender: Mixed	Not reported	FLO: 66.3 (4.62) SFO: 68.0 (5.69)	RCT^a^	FLO/SFO	NA	30 ml/d (ALA: 24 g)	NA	13 weeks/90 days
Brahe et al. [44]	Denmark	35/40	Obese postmenopausal women; BMI: 30–45 kg/m^2^; Gender: Woman	Hyperlipidemia TC (6.36 ± 0.89, 6.13 ± 1.06) mmol/L; LDL-C (4.11 ± 0.84, 3.73 ± 0.84) mmol/L	Flax: 60.6 (6.4) Con:5 8.5 (5.3)	RCT^a^	Flaxseed mucilage/ Placebo buns	10 g	NA	NA	6 weeks
Machado et al. [22]	Brazil	61/75	Overweight adolescents; BMI: (Flax: 23.38 ± 2.33, Co: 23.71 ± 2.01) kg/m^2^; Gender: Mixed	Non dyslipidemia	13.7 (2.1)	RCT^a^	Brown flaxseed/wheat bran	28 g	NA	NA	11 weeks
							Golden flaxseed/wheat bran				
Hutchins et al. [21]	US	25/41	Overweight or obese men and postmenopausal women; BMI: 30.4 ± 5.3 kg/m^2^; Gender: Mixed	Not reported	Men: 56.9 (8.3) Women: 60.0 (4.1)	RCT^b^	Flaxseed powder/non- flaxseed	13 g	NA	NA	12 weeks
								26 g			
Rhee et al. [33]	US	9/11	Obese glucose intolerant people; BMI (Flax: 32.4 ± 8.2, Con: 32.0 ± 8.3) kg/m^2^; Gender: Mixed	Not reported	54.7 (6)	RCT^b^	Flaxseed powder/wheat bran	40 g	NA	NA	12 weeks
Faintuch et al. [43]	Brazil	18/20	Subjects with morbidly obese; BMI (44.0 ± 3.9, 45.2 ± 4.2) kg/m^2^; Gender: Mixed	Non dyslipidemia	Flax: 47.8 (8.0) Con: 50.7 (6.4)	RCT^a^	Flaxseed powder/cassava powder	60 g	NA	NA	12 weeks
Faintuch et al. [42]	Brazil	24/24	Subjects with morbidly obese; BMI (Flax: 47.1 ± 7.2, Con: 47.2 ± 7.2) kg/m^2^; Gender: Mixed	Non dyslipidemia	40.8 (11.6)	RCT^a^	Flaxseed flour/manioc flour	30 g	NA	NA	2 weeks

US, United States; UK, United Kingdom ; TC, total cholesterol; TG, triglyceride; LDL-C, low-density lipoprotein cholesterol; HDL-C, high-density lipoprotein cholesterol; FLO, flaxseed oil; FO, fish oil; CO, corn oil; HPO, hempseed oil; OO, olive oil; SFO, sunflower oil; SAO, safflower oil; SBO, soybean oil; HORO, high-oleic rapeseed oil; WF, whole flaxseed; LIG, flaxseed lignan; HOCO, novel high-oleic rapeseed (canola) oil; LC, lifestyle counseling; VE, Vitamin E; NAFLD, nonalcoholic fatty liver disease; T2DM, Type 2 diabetic mellitus; CHD, coronary heart disease; MetSyn, metabolic syndrome; WC, waist circumference

^a^a randomized clinical trial

^b^a randomized crossover study

Milled flaxseed (including brown flaxseed [40, 48, 49], golden flaxseed [22], roasted flaxseed powder [46], non-specific sources mentioned flaxseed [21, 23, 28, 30–34, 42–44, 50, 53], or defatted form [36]) was experimented in 19 trials with doses of 13 to 68 g/d. Wheat bran/germ, muffins, cassava powder, sunflower seed or regular diets were chosen as the control regimen in these studies. Flaxseed oil has been tested in 10 trials [29, 35, 37–39, 41, 45, 47, 51, 52, 54] with doses of 0.4 to 24 g/d. The control regimens included oils enriched in the monounsaturated fatty acids (MUFAs) such as soybean oil or/and in the polyunsaturated fatty acids (PUFAs) such as safflower oil, corn oil or sunflower oil. In the remaining 2 trials [19, 20], flaxseed lignin intervention was used with doses from 0.02 to 0.6 g, and the controls were assigned to placebo (corn starch, or maltodextrin). These trials varied in length from 2 to 12 months. Twenty-six trials were designed as a parallel group and 5 as crossover studies [21, 32, 33, 35, 45].

Studies were performed in patients with different dyslipidemia related diseases: 2 studies were performed in patients with NAFLD [47, 48], 16 in patients with dyslipidemia [19, 20, 23, 30–32, 35–40, 45, 46, 50, 53], 7 in patents with overweight or obesity [21, 22, 29, 33, 41–44], 3 in patients with MetSyn [28, 49, 52], and 2 in patients with atherosclerosis [34, 51]. According to the classification of dyslipidemia status, 4 studies were performed in patients with hypertriglyceridemia [45, 47–49], 7 in patients with hypercholesterolemia [19, 20, 23, 30–32, 35], 10 in patients with multiple dyslipidemia states [28, 36–40, 44, 46, 50, 53], and 6 in patients with non-dyslipidemia [22, 29, 34, 42, 43, 51]. Moreover, 14 studies were performed in patients with BMI 25- < 30 kg/m^2^ [19, 23, 28, 29, 32, 35, 37–39, 45–47, 50, 53], and 10 in patients with BMI ≥ 30 kg/m^2^ [21, 33, 40–44, 48, 49, 52].

### Quality assessment

Six RCTs indicated the method for random sequence generation was low risk [29, 34, 44, 47, 48, 52], three other studies indicated high risk [21, 41, 53], and the remaining studies have unclear risk of bias. Allocation concealment was reported in 15 trials [20, 22, 23, 29, 32, 33, 35, 37, 40–42, 44, 45, 47, 51], 4 of these were regarded as high risk [46, 48, 50, 52], and the remaining trials indicated unclear risk of bias. Seventeen studies mentioned whether to apply double-blinding [19, 22, 23, 29–31, 34, 35, 37, 38, 41–45, 47, 51, 54], the 12 studies showed unclear risk of bias, whereas 5 indicated high risk [32, 35, 40, 48, 53]. Blinding of outcome assessment was reported in 10 studies as low risk [21, 35, 39, 41–45, 49, 51], 4 demonstrated high risk of bias [46, 50, 52, 53] and the remaining studies showed unclear risk of bias. All studies showed low risk of bias in terms of incomplete outcome data and unclear of selective reporting. The systematic assessment of bias is shown in Table 3.

**Table 3:** Risk of bias assessment of the included articles

Authors/study	Random sequence generation (selection bias)	Allocation sequence concealment (selection bias)	Blinding of participants and personnel (performance bias)	Blinding of outcome assessment (detection bias)	Incomplete outcome data (attrition bias)	Selective outcome reporting (reporting bias)
Rezaei et al. [47]	L	L	L	U	L	U
Raygan et al. [51]	L	L	L	L	L	L
Yang et al. [29]	L	L	L	U	L	U
Edel et al. [34]	L	U	L	U	L	L
Akrami et al. [52]	L	H	U	H	L	U
de Oliveira et al. [41]	H	L	L	L	L	U
Yari et al. [48]	L	H	H	U	L	U
Yari et al. [49]	U	U	U	L	L	U
Cassani et al. [40]	U	L	H	U	L	U
Machado et al. [22]	U	L	L	U	L	U
Brahe et al. [44]	L	L	L	L	L	U
Torkan et al. [50]	U	H	U	H	L	L
Saxena et al. [46]	U	H	U	H	U	L
Dittrich et al. [45]	U	L	L	L	L	U
Soltani et al. [53]	H	U	H	H	L	L
Hutchins et al. [21]	H	U	U	L	L	U
Faintuch et al. [43]	U	L	L	L	L	U
Gillingham et al. [35]	L	L	H	L	L	U
Rhee et al. [33]	U	L	U	U	L	U
Fukumitsu et al. [20]	U	U	L	U	L	L
Wu et al. [28]	U	H	U	U	U	U
Bloedon et al. [23]	U	L	L	U	L	L
Patade et al. [30]	U	U	L	U	L	L
Zhang et al. [19]	U	U	L	U	L	L
Faintuch et al. [42]	U	L	L	L	L	U
Paschos et al. [37]	U	L	L	U	L	U
Rallidis et al. [39]	U	U	U	L	L	U
Rallidis et al. [38]	U	U	L	U	L	L
Jenkins et al. [36]	U	U	U	U	L	L
Arjmandi et al., [31]	U	U	L	U	L	L
Bierenbaum et al. [32]	U	L	H	U	L	U

H, high risk; L, low risk; U, unclear

## Meta-analysis results

### Flaxseed supplementation on lipid profiles in patients with dyslipidemia-related diseases

#### Flaxseed supplementation on TC

**Fig. 1:**
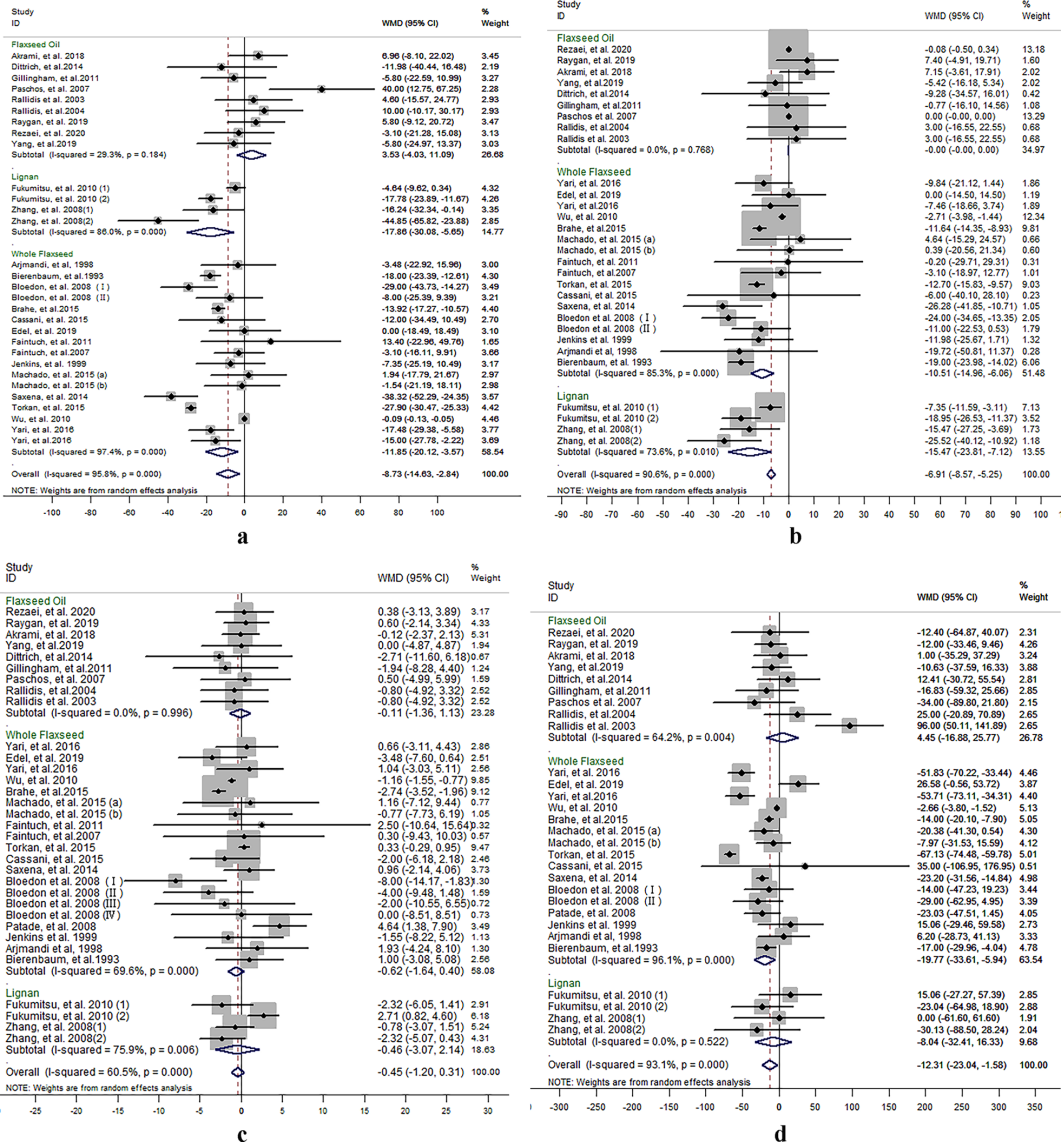
Effect of different flaxseed products on net changes (95% CI) of **a** TC, **b** LDL-C, **c** HDL-C and **d** TG in patients with dyslipidemia related diseases; (1) and (2) represent the low dose and high dose in two studies (*Zhang *et al*. and Fukumitsu *et al*.* respectively); **a** and **b** represent brown and golden flaxseed interventions respectively in the study by *Machado *et al*.*; (I) and (II) represent short and long intervention time respectively in the study by *Bloedon *et al.

The results for TC were reported in 30 comparisons from 26 studies including 1,562 participants. Meta-analysis suggested a significant net change in TC concentrations following supplementation with flaxseed-containing products (WMD − 8.73 mg/dL, 95% CI − 14.63, − 2.84, *P* = 0.004; *I*^2^ = 95.8%) compared to control groups, in which TC concentrations were decreased more significantly in studies using whole flaxseed and lignan supplementation (whole flaxseed: − 11.85 mg/dL, 95% CI − 20.12, − 3.57, *P* = 0.005; *I*^2^ = 97.4%; lignan: − 17.86 mg/dL, 95% CI − 30.08, − 5.65, *P* = 0.004; *I*^2^ = 86.0%), rather not using flaxseed oil (shown in Fig. 1a).

There was high level of heterogeneity among the studies that explored effects of flaxseed products on serum TC (*I*^2^ = 95.8%). Meta-regression was conducted to explore the heterogeneity, and found that the heterogeneity could be explained by type of flaxseed products (*P* = 0.01) (shown in Additional file 3: Table 1).

Egger’s linear regression (TC: intercept = − 2.16, *P* = 0.014) suggested publication bias in the meta-analysis.

#### Flaxseed supplementation on LDL-C, HDL-C and TC/HDL-C

Concentrations of LDL-C were reported in 30 comparisons from 26 studies representing 1,559 participants. Meta-analysis suggested significant net change in LDL-C concentrations following supplementation with flaxseed-containing products (WMD = − 6.92 mg/dL, 95% CI − 8.58, − 5.25; I^2^ = 90.6%), in which whole flaxseed and lignan supplementation had significant lowering-effect on LDL-C concentrations, rather than flaxseed oil intervention (shown in Fig. 1b).

In 27 articles, there are 33 comparisons included 1,596 participants reported effect of flaxseed supplementation on HDL-C concentrations. Meta-analysis suggested no significant net change in HDL-C following supplementation with flaxseed-containing products (WMD = − 0.45 mg/dL, 95% CI − 1.20, 0.31; I^2^ = 60.5%) (shown in Fig. 1c).

**Fig. 2:**
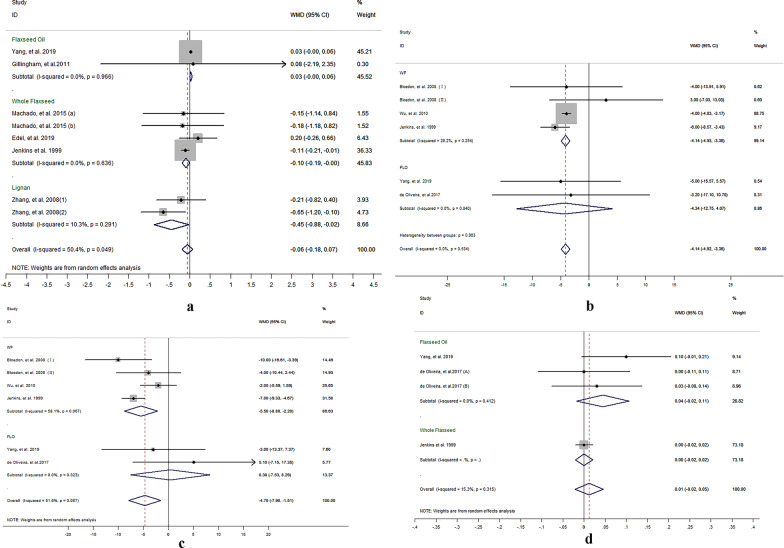
Effect of different flaxseed products on net changes (95% CI) of **a** TC/HDL-C, **b** apo A, **c** apo B and **d** apo A/apo B in patients with dyslipidemia related diseases; (1) and (2) represent the low dose and high dose in two studies (*Zhang *et al*. and Fukumitsu *et al*.* respectively); **a** and **b** represent brown and golden flaxseed interventions respectively in the study by *Machado *et al*.*; (I) and (II) represent short and long intervention time respectively in the study by *Bloedon *et al.

Eight comparisons included 378 participants from 6 studies reported the results for TC/HDL-C ration. Meta-analysis results suggested there was no significant net change in TC/HDL-C ration following supplementation with flaxseed-containing products (WMD = − 0.06, 95% CI − 0.18, 0.07; I^2^ = 50.4%). But subgroup analysis on whole flaxseed and lignan supplementation respectively showed mild net change on TC/HDL-C ration with significant difference (whole flaxseed: − 0.10, 95% CI − 0.19, − 0.00; I^2^ = 0.0%, and lignan: − 0.45, 95% CI − 0.88, − 0.02; I^2^ = 10.3%) (shown in Fig. 2a).

High level of heterogeneity was found among the studies that explored effects of flaxseed products on serum LDL-C (I^2^ = 95.7%) and HDL-C (I^2^ = 92.0%). Meta-regression was conducted to explore the heterogeneity, and found that the heterogeneity of LDL-C related studies could be explained by factors that were lipid status (*P* = 0.013) and type of flaxseed products (*P* = 0.001), the heterogeneity of HDL-C related studies could be explained by intervention time (*P* = 0.036) (shown in Additional file 3: Table 1).

Egger’s linear regression revealed a potential publication bias in the meta-analysis of flaxseed’s effects on plasma LDL-C concentrations (LDL-C: intercept = − 1.97, *P* < 0.01), rather than HDL-C concentrations (LDL-C: intercept = 0.28, *P* = 0.423).

#### Flaxseed supplementation on TG

Twenty-nine comparisons from 24 studies reported the results for TG, which included 1,552 participants. The pooled result suggested that there was a significant net change in TG following supplementation with flaxseed-containing products (WMD − 12.31 mg/dL, 95% CI − 23.04, − 1.58, *P* = 0.025; *I*^2^ = 93.1%). Whole flaxseed supplementation had a significant effect on reducing TG concentration (WMD − 19.77 mg/dL, 95% CI − 33.61, − 5.94,* P* = 0.005; *I*^2^ = 96.1%) compared to control group according to the meta-analysis for different type of flaxseed products (shown in Fig. 1d).

There was high level of heterogeneity among the studies that explored effects of flaxseed products on serum TG (*I*^2^ = 93.1%). Meta-regression was conducted to explore the heterogeneity, and found that the heterogeneity of TG related studies could be explained by lipid status (*P* = 0.044) and country factor (*P* = 0.041) (shown in Additional file 3: Table 1).

Egger’s linear regression (TG: intercept = − 1.167, *P* = 0.137) suggested no publication bias in the meta-analysis.

#### Flaxseed supplementation on Apo A, Apo B and Apo B/Apo A

Six comparisons from 5 studies reported the results for Apo A and Apo B, which referring to 440 participants. Results suggested that there was a significant reduction in Apo A and Apo B following supplementation with flaxseed-containing products (Apo A: − 4.14 mg/dL, 95% CI − 4.92, − 3.36; *I*^2^ = 0%; Apo B: − 4.70 mg/dL, 95% CI − 7.90, − 1.51; *I*^2^ = 51.6%;). Thereinto, whole flaxseed supplementation has significant effects on apolipoproteins (Apo A: − 4.14 mg/dL, 95% CI − 4.92, − 3.36; *I*^2^ = 26.2%; Apo B: − 5.50 mg/dL, 95% CI − 8.80, − 2.20; *I*^2^ = 58.1%;), but flaxseed oil intervention had no such effect (Apo A: − 4.34 mg/dL, 95% CI − 12.75, 4.07; *I*^2^ = 0%; Apo B: 0.38 mg/dL, 95% CI − 7.53, 8.29; *I*^2^ = 0%;). (shown in Fig. 2b, c).

Three comparisons included 189 participants from 3 studies reported the results for Apo B/Apo A ration. Meta-analysis revealed no significant effect of flaxseed supplementation on Apo B/ Apo A ration (Apo B/Apo A: 0.03, 95% CI − 0.03, 0.08; *I*^2^ = 43.4%; *P* = 0.372), no matter whole flaxseed intervention or flaxseed oil (Fig. 2d).

Egger’s linear regression suggested no publication bias in the meta-analysis of flaxseed supplementation on Apo A, Apo B and Apo B/Apo A (Apo A: intercept = 0.09, *P* = 0.874; Apo B: intercept = 1.10, *P* = 0.389; Apo B/Apo A: intercept = 1.49, *P* = 0.327).

### Flaxseed supplementation on inflammatory cytokines in patients with dyslipidemia-related diseases

#### Flaxseed supplementation on IL-6

**Fig. 3:**
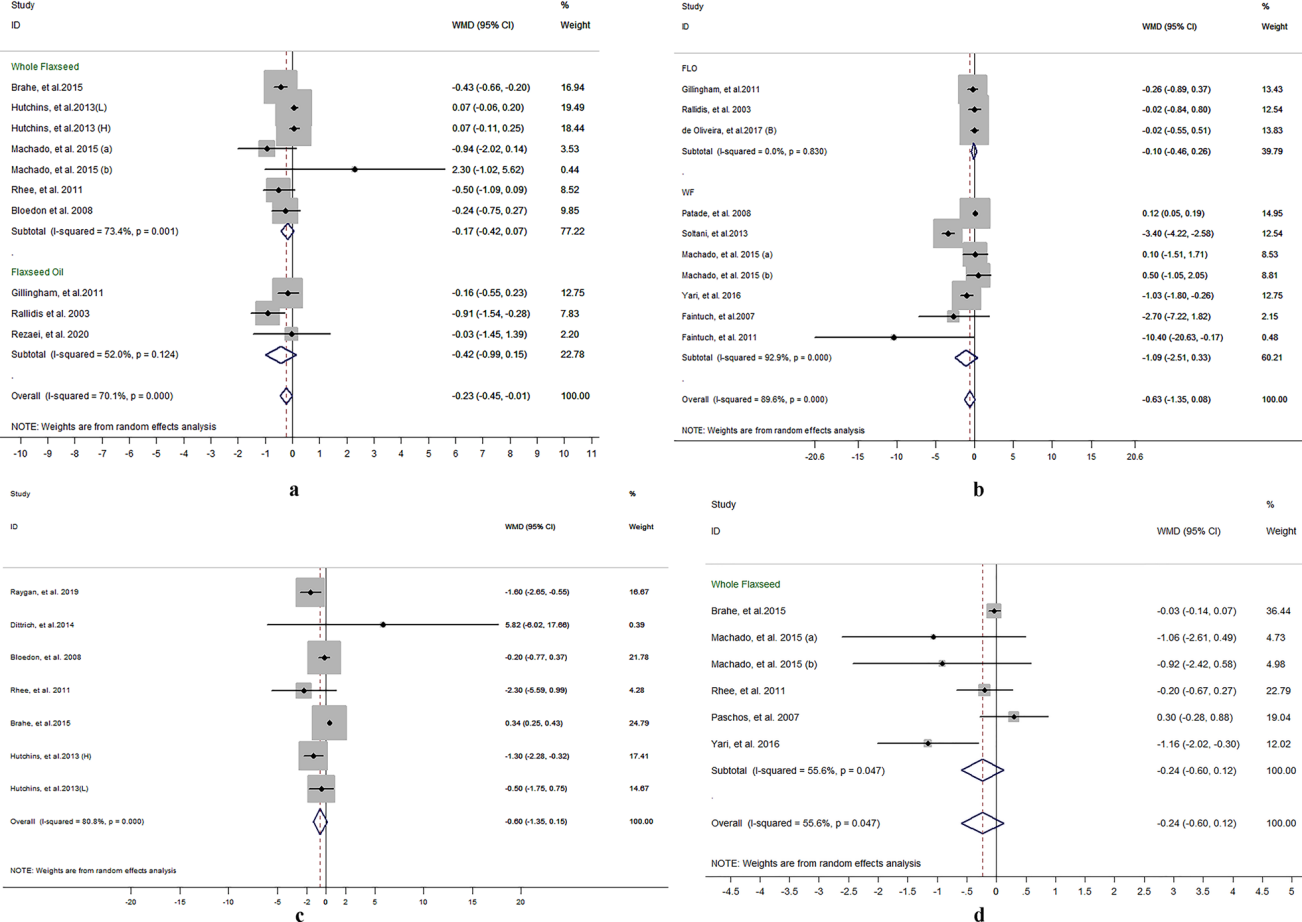
Effect of different flaxseed products on net changes (95% CI) of **a** IL-6, **b** CRP, **c** hs-CRP and **d** TNF-α in patients with dyslipidemia related diseases; **a** and **b** represent brown and golden flaxseed interventions respectively in the study by *Machado *et al*.*; (L) and (H) represent the low dose and high dose in respectively in the study by *Hutchins *et al.

The results for IL-6 were reported in 10 comparisons including 426 patients with dyslipidemia-related diseases. The pooled result showed a significant net change for IL-6 (WMD − 0.23 pg/mL, 95% CI − 0.45, − 0.01; *I*^2^ = 70.1%) in flaxseed supplementation compared to control group. Subgroup analysis revealed no effectiveness of whole flaxseed supplementation on IL-6 (WMD -0.17 pg/mL, 95% CI − 0.42, 0.07; *I*^2^ = 73.4%); but revealed a significant effect of flaxseed oil on IL-6 (WMD − 0.35 pg/mL, 95% CI − 0.67, − 0.03; I^2^ = 52.0%; *P* = 0.033) (shown in Fig. 3a).

There is moderate level of heterogeneity among the studies. The heterogeneity among the studies could be explained by study design (*P* = 0.006) in the meta-regression (shown in Additional file 3: Table 1).

Egger’s linear regression (intercept = − 1.25, *P* = 0.18) suggested no publication bias in the meta-analysis.

#### Flaxseed supplementation on CRP and hs-CRP

Seven comparisons included 284 participants from 6 studies reported the results for hs-CRP, and 11 comparisons included 443 participants from 9 studies reported CRP. Meta-analysis revealed no significant effect of supplementation with flaxseed-containing products on CRP (WMD − 0.63 mg/L, 95% CI − 1.35, 0.08; *I*^2^ = 89.6%; *P* = 0.083) and hs-CRP (WMD − 0.60 mg/L, 95% CI − 1.35, 0.15; *I*^2^ = 80.8%; *P* = 0.115) (shown in Fig. 3b, c). Subgroup analysis revealed separate consumption with whole flaxseed or flaxseed oil did not reduced CRP, however, the analysis showed flaxseed oil consumption reduced hs-CRP (WMD − 1.54 mg/L, 95% CI − 2.59, − 0.49; *I*^2^ = 33.1%; *P* = 0.004).

The source of high level of heterogeneity was explained by country factor (*P* = 0.009) in the meta-regression of flaxseed intervention on CRP, but no factor could explain the heterogeneity in the meta-regression of flaxseed intervention on hs-CRP (shown in Additional file 3: Table 1).

Egger’s linear regression suggested no publication bias in the meta-analysis (CRP: intercept = − 1.90, *P* = 0.09; hs-CRP: intercept = − 1.86, *P* = 0.06).

#### Flaxseed supplementation on TNF-α

The protective effect of flaxseed supplementation was not seen in the analysis of TNF-α (WMD − 0.24, 95% CI − 0.24, 0.12; *I*^2^ = 55.6%) in 202 participants in 6 comparisons (from 5 studies) (Fig. 3d).

There is moderate level of heterogeneity among the studies. The heterogeneity among the studies could not be explained by presupposed factors in the meta-regression (shown in Additional file 3: Table 1).

Egger’s linear regression (intercept = − 1.126, *P* = 0.180) suggested no publication bias in the meta-analysis.

### Flaxseed supplementation on anthropometric indices in patients with dyslipidemia-related diseases

#### Flaxseed supplementation on weight and BMI

**Fig. 4:**
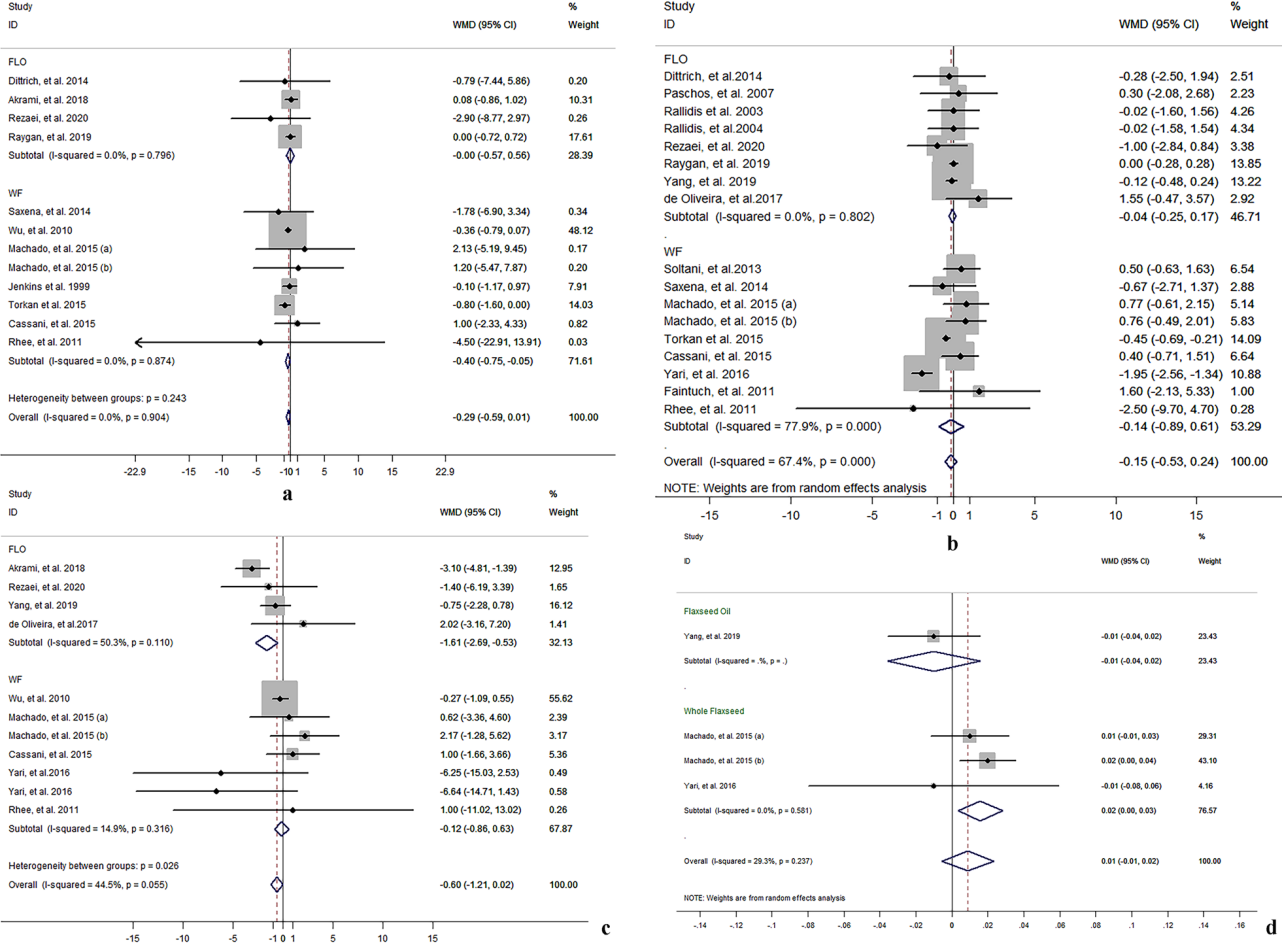
Effect of different flaxseed products on net changes (95% CI) of **a** weight, **b** BMI, **c** WC and **d** WHR in patients with dyslipidemia related diseases; **a** and **b** represent brown and golden flaxseed interventions respectively in the study by *Machado *et al.

The results for weight were reported in 12 comparisons from 11 studies including 706 participants. The pooled result from these studies showed no significant effect of supplementation with flaxseed-containing products on weight change (WMD − 0.29 kg, 95% CI − 0.59 0.01; *I*^2^ = 0.0%) (shown in Fig. 4a). But subgroup analysis revealed whole flaxseed supplementation significantly reduced the net weight change (WMD − 0.40 kg, 95% CI − 0.75, − 0.05; *I*^2^ = 0.0%) compared with the control group, while flaxseed oil supplementation did not (*P* = 0.991).

Seventeen comparisons included 949 participants from 16 studies reported the results for net change on BMI value (shown in Fig. 4b). Meta-analysis revealed no significant effect of supplementation with flaxseed-containing products on net change of BMI (WMD − 0.15 kg/m^2^, 95% CI − 0.53, 0.24; *I*^2^ = 67.4%), no matter whole flaxseed or flaxseed oil.

High level of heterogeneity was found among the studies that explored effects of flaxseed products on BMI (*I*^2^ = 67.4%). The high level of heterogeneity among the studies was explained by lipid status (*P* = 0.001) and study country (*P* = 0.047) in the meta-regression (shown in Additional file 3: Table 1).

Egger’s linear regression suggested no publication bias in the meta-analysis of flaxseed supplementation on weight and BMI value (weight: intercept = 0.02, *P* = 0.948; BMI: intercept = 0.42, *P* = 0.503).

#### Flaxseed supplementation on waist circumference (WC) and waist-height ration (WHR)

Eleven comparisons from 10 studies reported the results for WC, which involved 621 participants. The pooled analysis suggested that there was no significant net change in WC following supplementation with flaxseed-containing products (WMD − 0.60 cm, 95% CI − 1.21, 0.02; *I*^2^ = 44.5%) (shown in Fig. 4c). However, subgroup analysis revealed consumption with flaxseed oil had a significant reduction in net change for WC (WMD − 1.61 cm, 95% CI − 2.69, − 0.53; *I*^2^ = 50.3%) compared to control group.

Four comparisons included 200 participants from 3 studies reported the results for net change for WHR. Meta-analysis revealed no net change on WHR following supplementation with flaxseed-containing products (WMD 0.01, 95% CI − 0.01, 0.02; *I*^2^ = 29.3%) (shown in Fig. 4d); But subgroup results revealed separate consumption with whole flaxseed had an increase on net change for WHR (WMD 0.02, 95% CI 0.00, 0.03; *I*^2^ = 0.0%).

There is moderate level of heterogeneity among the studies that explored effects of flaxseed products on WC (*I*^2^ = 44.5%). The moderate level of heterogeneity among the studies was explained by study country (*P* = 0.047) in the meta-regression (shown in Additional file 3: Table 1).

Egger’s linear regression (WC: intercept = 0.161, *P* = 0.811; WHR: intercept = − 1.722, *P* = 0.331) suggested no publication bias in the meta-analysis of flaxseed supplementation on weight and BMI value.

#### Subgroup analysis

Further subgroup analyses were performed to explore impacts of certain characteristics. As the effect of whole flaxseed, flaxseed oil and lignans on the patients with lipid metabolism disorder is different, authors made subgroup analysis after stratifying pooled results according different type of flaxseed intervention. As the subgroups with less than two studies are not considered to be comparable, we did not compare differences within a subgroup with less than 2 studies of net changes. Detailed results of subgroup analysis are described in the Additional file 1 and Additional file 4: Table 2.

### Meta-regression analysis for whole flaxseed supplementation on lipid profiles, inflammatory cytokines and anthropometric indices

**Fig. 5:**
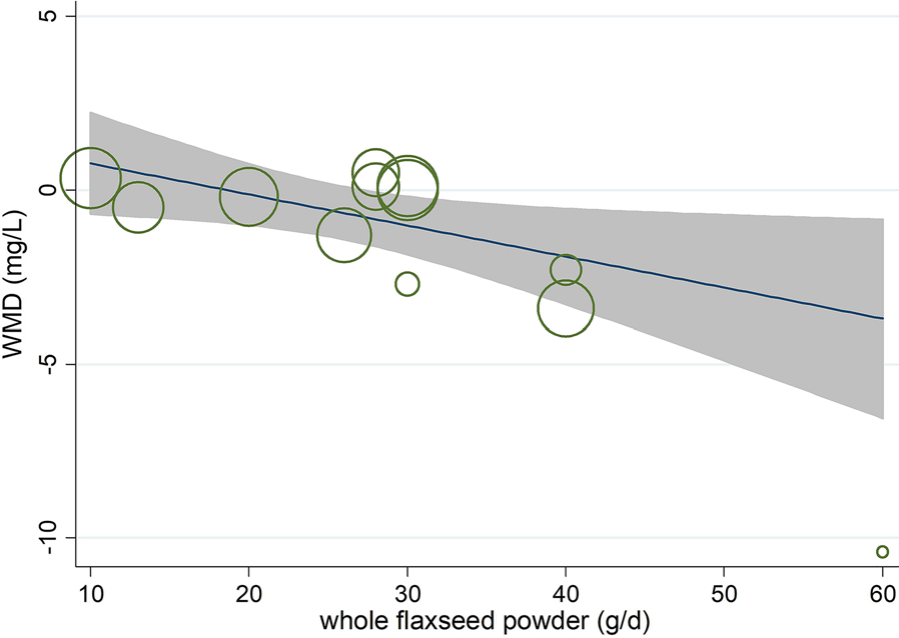
Random-effects meta-regression plot of the association between dose of whole flaxseed (g/day) and weighted mean difference of C-reactive protein (CRP)

Meta-regression using the random-effects model was undertaken to investigate the potential association between a decrease in lipid profile, anthropometric indices, and inflammatory cytokines and dose of whole flaxseed powder (g/day). It indicated a linear relationship between dose and absolute changes in CRP (*P* = 0.036) (shown in Fig. 5), but not in lipid profile (TC: *P* = 0.252; LDL-C: *P* = 0.327; HDL-C: *P* = 0.279; TG: *P* = 0.961), anthropometric indices (weight: *P* = 0.412; BMI: *P* = 0.406, WC: *P* = 0.523), and other inflammatory cytokines (IL-6: *P* = 0.759; TNF-α:* P* = 0.935, hs-CRP: *P* = 0.176) (shown in Additional file 5: Table 3).

### Curvilinear regression for whole flaxseed supplementation and lipid profile, inflammatory cytokines and anthropometric indices

**Fig. 6:**
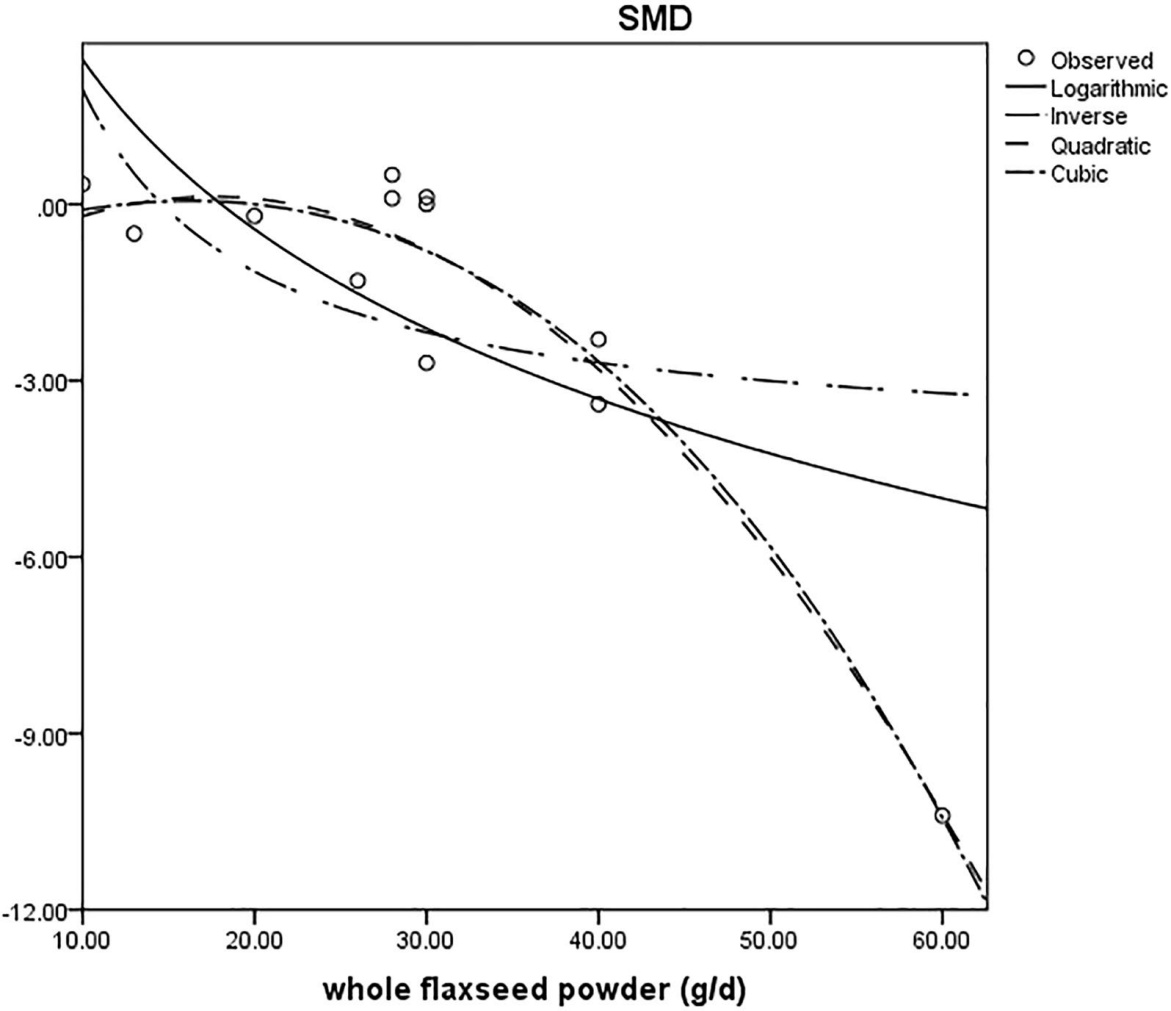
Curvilinear regression plot of the association between dose of whole flaxseed (g/day) and weighted mean difference of IL-6

Curvilinear regression was undertaken to investigate the potential association between a decrease in lipid profile, anthropometric indices, and inflammatory cytokines and dose of whole flaxseed powder (g/day). It indicated a nonlinear relationship between dose and absolute changes in IL-6 (*P* < 0.01; approximate Cubic formula: Y = − 0.897 + 0.109χ − 0.003χ^2^ − 3.169χ^3^) (shown in Fig. 6), but not in lipid profile, anthropometric indices, and other inflammatory cytokines (*P* > 0.05) (shown in Additional file 6: Table 4).

### Sensitivity analyses

Findings regarding serum lipid, inflammatory and anthropometric parameters basically remained robust in the sensitivity analysis when excluding a trial carrying a high risk of bias [53].

## Discussion

The present systematic review and meta-analysis revealed that flaxseed consumption had an overall beneficial effect on serum TC, LDL-C, TG, apo B and IL-6 in patients with dyslipidemia related diseases, but not on apo A, HDL-C, hs-CRP, CRP and anthropometric indices. Moreover, different flaxseed products (whole flaxseed, flaxseed oil and flaxseed lignan) had obviously different effects. Meta-analysis revealed a significant reduction on TC, LDL-C, TG, apo B, TC/HDL-C and weight loss in patients allocated to whole flaxseed; flaxseed lignan had a significant lowering effect on TC, LDL-C and TC/HDL-C; however, flaxseed oil showed a significant reduction on IL-6 and hs-CRP.

Our present results on TC, LDL-C and TG in patients with dyslipidemia related diseases were consistent with a meta-analysis [13] that studied on the whole population. Whole flaxseed was an essential source of high-quality protein and soluble fiber [54], which contained 41% fat, 20% protein, and 28% fiber [55]. Especially, dietary soluble of flaxseed has been reported repeatedly on its function of reducing blood lipid [56–58]. However, different bioactive components are present in different flaxseed-derived products [59], our meta-analysis revealed flaxseed oil had no such protective effect compared with whole flaxseed or lignans. It could be an explanation for more significant effects of whole flaxseed than form of flaxseed oil, that whole flaxseed is a combination of multiple nutrients including not only α-linolenic acid (ALA), but also phytoestrogen, and lignans, together with high-quality protein and soluble fiber which could result in synergistic interactions [60]. Another reason was that the cholesterol-lowering effects of flaxseed may be enhanced by baking, perhaps because of the effect of high temperature on the bioavailability of some of the flaxseed phytochemicals [30]. Additionally, flaxseeds are the best source of lignans (0.7–1.5%, secoisolariciresinol diglucoside [SDG]) [2, 61]. Flaxseed SDG has antioxidant, anti-inflammatory, and potent angiogenic and antiapoptotic properties, which plays a role in antiatherosclerosis [62]. Another possible explanation was that the effects of flaxseed oil may have been masked by the use of MUFA- or n-6 PUFA–enriched oils as the control regimen in these included studies [63].

Subgroups analysis after stratifying pooled results according different type of flaxseed intervention (as shown in Table 3). Subgroup analysis revealed that whole flaxseed had significant effects on the net reductions in TC, LDL-C, TG and apo B in patients with dyslipidemia and with abnormal weight. One of the plausible explanations for the findings is that patients with dyslipidemia were more sensitive to lipid-lowering products than healthy adults [64]. Moreover, apo (B)-lowering effects of whole flaxseed intervention were shown in present meta-analysis. Apolipoprotein B is an essential part of the very-low-density lipoprotein (VLDL) and low-density lipoprotein (LDL), which facilitates their binding to receptors. As previous studies observed, the changes in apo B are consistent with the decrease in LDL cholesterol [65, 66]. Flaxseed is the richest plant source of SDG, which is one of the three major groups of phytoestrogens. The effects of estrogen and selective estrogen receptor modulators such as Tamoxifen on reduction in lipoprotein (a) have been explored in many experimental and human studies [67, 68]. In this regard, phytoestrogens could increase bile acid secretion, enhance thyroid function, and modify hepatic metabolism to play a role on the cholesterol-lowering effect [69]. Interestingly, whole flaxseed decreased TC, LDL-C and TG levels irrespective of country and the intervention time prescribed, but was more pronounced when the dose of whole flaxseed was ≤ 30 g/day (TC: WMD − 13.61 mg/mL; LDL-C: WMD − 10.52 mg/mL; TG: WMD − 23.52 mg/mL), rather not a dose > 30 g/day. Our findings confirmed excessive dietary intake may not bring more health benefits. Consistently, one ounce (28 g) of flaxseed exceeds the Adequate Intake (AIs) for ALA, which is 1.1 g/day for women and 1.6 g/day for men, and 1.4 g/day and 1.3 g/day during pregnancy and lactation, respectively [70–72].

We did not observe any significant effects of flaxseed or its derivatives on HDL cholesterol. These findings were completely consistent with that meta-analysis [13, 63]. The results of most current, current studies still support this view [65, 66, 73, 74]. Apo A1 is the primary protein component of HDL that plays a role in HDL metabolism [75]. Actually, the apo (A)-lowering effects of whole flaxseed intervention were revealed in our meta-analysis, and this result well explained that flaxseed intervention had no significant effect on HDL-C. However, HDL-C without increase seems be not a negative effect on patients with lipid metabolism. A recent register study of more than 1 million US veterans found a U-shaped relationship between HDL-C and total mortality with 50 mg/dL (1.25 mmol/L) as the nadir associated with the lowest mortality [76]. We carefully reviewed that the baseline HDL-C of the included population was basically maintained at about 1.25 mmol/L. Similar to us, a retrospective showed that very high concentrations of HDL-C might be not associated with decreased risk [77]. Secondly, several studies also have provided evidence that the vascular effects of HDL are variable and hardly correlate with HDL-C concentrations in the plasma. In patients with diabetes mellitus, coronary disease, chronic renal insufficiency, NAFLD, cardiovascular risk factors and disorders, the function of HDL is impaired [78, 79].

Previous meta-analysis assessed the effects of flaxseed and flaxseed-derived products (flaxseed oil or lignans) on inflammatory cytokines, did not reach consistent conclusions [15, 80, 81]. The present meta-analysis indicated that flaxseed supplementation significantly decreased IL-6, rather not CRP, hs-CRP and TNF-α. Our meta-analysis was conducted in a specific population with dyslipidemia related diseases. As some studies suggested these patients with metabolic disorders may get more benefits from the intervention, for obese and unhealthy subjects tending to accompany with higher levels of inflammatory factors [82, 83]; for instance, CRP is an indicator of general low-grade inflammation [84, 85]. Flaxseed related active compounds, especially SDG and its metabolites, have shown anti-inflammatory and antioxidant activity, mainly through inhibition of lipid peroxidation. A study [52] revealed that flaxseed oil mainly plays roles in antioxidant activity to reduce oxidation of low-density lipoprotein cholesterol (LDL-C), rather than its concentrations. In accordance with this, the present study revealed flaxseed oil had the lowering-effect on IL-6 and hs-CRP, rather not lipid profiles. Flaxseed oil mainly contains α-linolenic acid (ALA), ALA competes with linoleic acid (LA) in their common metabolism pathways for producing their long-chain metabolites. It could decrease production of arachidonic acid (AA) and consequently decreased appearance of this fatty acid in tissues has been suggested to decrease proinflammatory eicosanoid, which may decrease inflammation [81]. Meta-regression revealed a linear relationship between dose of whole flaxseed consumption and absolute changes in CRP and a nonlinear relationship between with IL-6. In fact, interactions between diet, inflammation, and the microbiota should be considered [86]. Flaxseed is one of the richest sources of soluble fiber which has been shown to be fermented to short-chain fatty acid (SCFA) (acetate, propionate, and butyrate) by intestinal bacteria. SCFA, especially propionate, can plays as anti-inflammatory factors through interference in various inflammatory pathways [87].

Our meta-analysis found whole flaxseed consumption had a significant reduction on net change for weight (WMD = − 0.40 kg;* P* = 0.027; I^2^ = 0.0%) compared to control group. Although we did not find its significant reduction on BMI value (*P* = 0.719), however, subgroup result suggested its significant net change in participants with dyslipidemia (n = 6, WMD = − 0.31, *P* = 0.007). These results suggest that flaxseed has a beneficial effect on anthropometric indices. Whole flaxseed rather than flaxseed-oil seems effective in weight and BMI reduction which is attributed to the fact that the flaxseed can control the energy intake and increase satiety by containing 28% fibers [88]. These findings also could explain by the subsequent increase in circulating ALA after flaxseed supplementation. The anti-obesity effects of eicosapentaenoic acid and docosahexaenoic acid have been shown in previous studies [89, 90].

### Strengths and limitations

The strengths of the current study were that it was the first to explore the effect of flaxseed on lipid profiles, inflammatory cytokines and anthropometric indices in patients with dyslipidemia related diseases, and to distinguish the different effects of different types of flaxseed products. In addition, authors made subgroups analysis after stratifying pooled results according different type of flaxseed intervention to find the differences between subgroups more persuasively. It is more noteworthy that this study found revealed a linear or nonlinear relationship between dose of whole flaxseed consumption and absolute changes in inflammatory factors. Although our meta-analysis provides stronger evidence on the function of flaxseed interventions, these findings must be cautioned to applicate for the reasons as following: (1) the most of SDs of the net change were estimated using correlation coefficient methods referenced in the Cochrane Handbook, rather than were exact value; (2) many characteristics that vary within studies could not remove to be factors of between-study heterogeneity, such as difference of the different background of the patients included, the varying control groups, or the uneven quality of the studies; (3) some relevant studies might be missing from our pooling because neither grey literature databases were used for identifying studies, nor non-English studies were considered; (4) influences of the other covariates could not be fully determined because of the lack of information of the quality of products and the amount of specific bioactive components in flaxseed and their bioavailability. Further studies are needed with large sample sizes, adequate durations, and solid study designs to investigate the effectiveness of flaxseed supplementation.

## Conclusions

All in all, flaxseed intervention suggested the positive effects on lipid profiles, inflammatory cytokines and anthropometric indices in patients with dyslipidemia related diseases. Of these, whole flaxseed and lignans play an important role in reducing blood lipid, while flaxseed oil mainly plays in anti-inflammatory. Lipid- and weight-lowering was significant when whole flaxseed was consumed at doses < 30 mg/d, for lipid status with mixed dyslipidemia and patients with BMI > 25. Additionally, a linear association between whole flaxseed consumption and absolute changes in C-reactive protein and a nonlinear relationship between with IL-6 was observed.

## Supplementary Information

**Additional file 1**. Subgroup analysis of flaxseed supplementation on lipid profiles, inflammatory cytokines and anthropometric indices in patients with dyslipidemia related diseases.

**Additional file 2**. The flow diagram of systematic review and meta-analysis.

**Additional file 3**. Meta-regression investigating the associations between flaxseed-derived product intakes and lipid profiles, inflammatory factors and anthropometric indices.

**Additional file 4**. Subgroup analysis on the effects of flaxseed powder and flaxseed oil on lipid profiles, inflammatory cytokines and anthropometric indices in patients with dyslipidemia related diseases.

**Additional file 5**. Meta-regression on linear relationship between dose and absolute changes in lipid profiles, inflammatory cytokines and anthropometric indices.

**Additional file 6**. Curvilinear regression on nonlinear relationship between dose of whole flaxseed powder and absolute changes inlipid profiles, inflammatory cytokines and anthropometric indices.

## Data Availability

The tables, figures and supplemental materials supporting the conclusions of this article are included within the article.

## Abbreviations

WMD: Weighted mean difference
CI: Confidence interval
TC: Total cholesterol
TG: Triglyceride
LDL-C: Low-density lipoprotein cholesterol
HDL-C: High-density lipoprotein cholesterol
apo A: Apolipoprotein A
apo B: Apolipoprotein B
hs-CRP: High sensitivity C-reactive protein
BMI: Body mass index
WC: Waist circumference
MetSyn: Metabolic syndrome
NAFLD: Nonalcoholic fatty liver disease
ALA: α-Linolenic acid
LA: Linoleic acid
WF: Whole flaxseed
LIG: Flaxseed lignan
T2DM: Type 2 diabetic mellitus
CHD: Coronary heart disease
SCFA: Short-chain fatty acid
SDG: Secoisolariciresinol diglucoside
