# Supplementary material for: Comparisons of the effects of different flaxseed products consumption on lipid profiles, inflammatory cytokines and anthropometric indices in patients with dyslipidemia related diseases: systematic review and a dose–response meta-analysis of randomized controlled trials
Source: Nutr Metab (Lond). 2021 Oct 11;18:91. doi: 10.1186/s12986-021-00619-3 (PMC8504108; doi:10.1186/s12986-021-00619-3)
Supplement: Supplementary file 1 — Additional file 1. Subgroup analysis of flaxseed supplementation on lipid profiles, inflammatory cytokines and anthropometric indices in patients with dyslipidemia related diseases. [file 12986_2021_619_MOESM1_ESM.docx]

**Subgroup Analysis**

1. **Subgroup Analysis of Flaxseed Supplementation on Lipid Profiles in Patients with Dyslipidemia-related Diseases**

Subgroup analysis revealed that whole flaxseed consumption significantly reduced serum TC and LDL-C concentrations in patients with BMI > 25 (TC: n = 6, WMD -19.99 mg/dL, *P* = 0.012, *I^2^* = 99.1%; LDL-C: n = 6, WMD -14.83 mg/dL, P < 0.001, *I^2^* = 94.2%) and patients with BMI ≥ 30 (TC: n = 6, WMD -13.41 mg/dL, *P* < 0.001, *I^2^* = 2.2%; LDL-C: n = 6, WMD -11.01 mg/ dL, *P* < 0.001, *I^2^* = 0%). Subgroup analysis on lipid status showed whole flaxseed intervention reduced the serum TC and LDL-C concentrations in patients with hypercholesterolemia, hypertriglyceridemia or mixed dyslipidemia, especially significant in hypercholesterolemic patients (*P _intergroup_* < 0.01). Moreover, subgroup analysis suggested whole flaxseed consumption reduced significantly the absolute changes in TC and TG concentrations with statistical difference (*P* < 0.05) in both Asian and Westerner (Asian *vs.* Westerner, TC: -19.42 mg/dL *vs.* -13.53 mg/dL; TG: -39.32 mg/dL *vs.* -11.52 mg/dL); and this between-subgroup difference was not statistically significant (*P _interaction_* > 0.05). Subgroup analysis also detected the significant net changes on TC and LDL-C concentrations, regardless participants allocating to intervention time with ≤ 10 weeks or > 10 weeks. In addition, subgroup analysis revealed there are more significant different in net changes of TC and LDL-C in patients with dose of whole flaxseed allocated to no more than 30 g/d, rather not in patients with dose of more than 30 g/d. Similar to the results of the above subgroup analysis, subgroup analysis revealed the whole flaxseed intervention significantly reduced apo B in patients with BMI > 25 (n = 3, WMD -3.86 mg/dL, *P* = 0.008), in patients with hypercholesterolemia (n = 2, WMD -6.92 mg/dL, *P* = 0.003), in Westerner (n = 3, WMD -6.98 mg/dL, *P* < 0.01), or intervention dose ≤ 30 g/d and intervention time ≤ 10 weeks.

For subgroup analysis on effects of flaxseed oil on lipid profiles, results revealed no significant net change for these indexes in patients allocated to any subgroup.

1. **Subgroup Analysis of Flaxseed Supplementation on Inflammatory Cytokines in Patients with Dyslipidemia-related Diseases**

As the subgroups with less than two studies are not considered to be comparable, we did not compare differences on the net changes of inflammatory after flaxseed oil consumption within subgroups stratified by BMI categories, gender, lipid status, country and dose factors. However, the net change of IL-6 in participants consumed with whole flaxseed in patients with mixed hyperlipidemia (n =3, WMD -0.44 pg/mL, 95% CI -0.67, -0.21, *P* < 0.001, *I^2^* = 42.1%), and participants allocated to intervention time with ≤ 10 weeks decreased more significantly (n =2, WMD -0.40 pg/mL, 95% CI -0.61, -0.19, *P* < 0.001, *I^2^* = 0.0%). In addition, subgroup analysis revealed whole flaxseed consumption had a significant reduction on CRP concentration in patients with BMI ≥ 30 (n = 3, WMD -1.13 mg/L, 95% CI -1.89, -0.37, *P* = 0.004), but increased CRP concentration in Westerner (n = 5, WMD 0.12 mg/L, 95% CI 0.05, 0.19, *P* = 0.001).

1. **Subgroup Analysis of Flaxseed Supplementation on Anthropometric Indices in Patients with Dyslipidemia-related Diseases**

Subgroup analysis on anthropometric index revealed that there is significant net change in weight on whole flaxseed intervention in patients with BMI ≥ 25 (n = 3, WMD -0.47 kg, 95% CI -0.85, -0.09, *P* = 0.016, *I^2^* = 0.0%), with mixed hyperlipidemia (n = 7, WMD -0.40 kg, 95% CI -0.75, -0.04, *P* = 0.028, *I^2^* = 0.0%) or Asian (n = 3, WMD -0.47 kg, 95% CI -0.85, -0.09, *P* = 0.016, *I^2^* = 0.0%) and dose ≤ 30 g/d subgroups (n = 5, WMD -0.45 kg, 95% CI -0.83, -0.08, *P* = 0.019, *I^2^* = 0.0%). For BMI, subgroup result suggested its significant reduction on net change in participants with BMI ≥ 25 (n = 3, WMD -0.41 kg, 95% CI -0.65, -0.17, *P* = 0.001, *I^2^* = 24.8%), or mixed hyperlipidemia (n = 6, WMD -0.31 kg, 95% CI -0.53, -0.08, *P* = 0.007, *I^2^* = 50.5%) and intervention time ≤ 10 weeks (n = 3, WMD -0.37, 95% CI -0.61, -0.14, *P* = 0.002, *I^2^* = 55.8%).

For subgroup analysis on effects of flaxseed oil on anthropometric index, subgroup results revealed no significant net change for these indexes in patients allocated to flaxseed oil intervention, excepting for WC on subgroup of Asian population (n = 3, WMD -1.78 cm, 95% CI -2.88, -0.67, *P* = 0.002, *I^2^* = 50.7%).
