# Supplementary material for: Comparisons of the effects of different flaxseed products consumption on lipid profiles, inflammatory cytokines and anthropometric indices in patients with dyslipidemia related diseases: systematic review and a dose–response meta-analysis of randomized controlled trials
Source: Nutr Metab (Lond). 2021 Oct 11;18:91. doi: 10.1186/s12986-021-00619-3 (PMC8504108; doi:10.1186/s12986-021-00619-3)
Supplement: Supplementary file 2 — Additional file 2. The flow diagram of systematic review and meta-analysis. [file 12986_2021_619_MOESM2_ESM.pdf]

PRISMA 2009 Flow Diagram

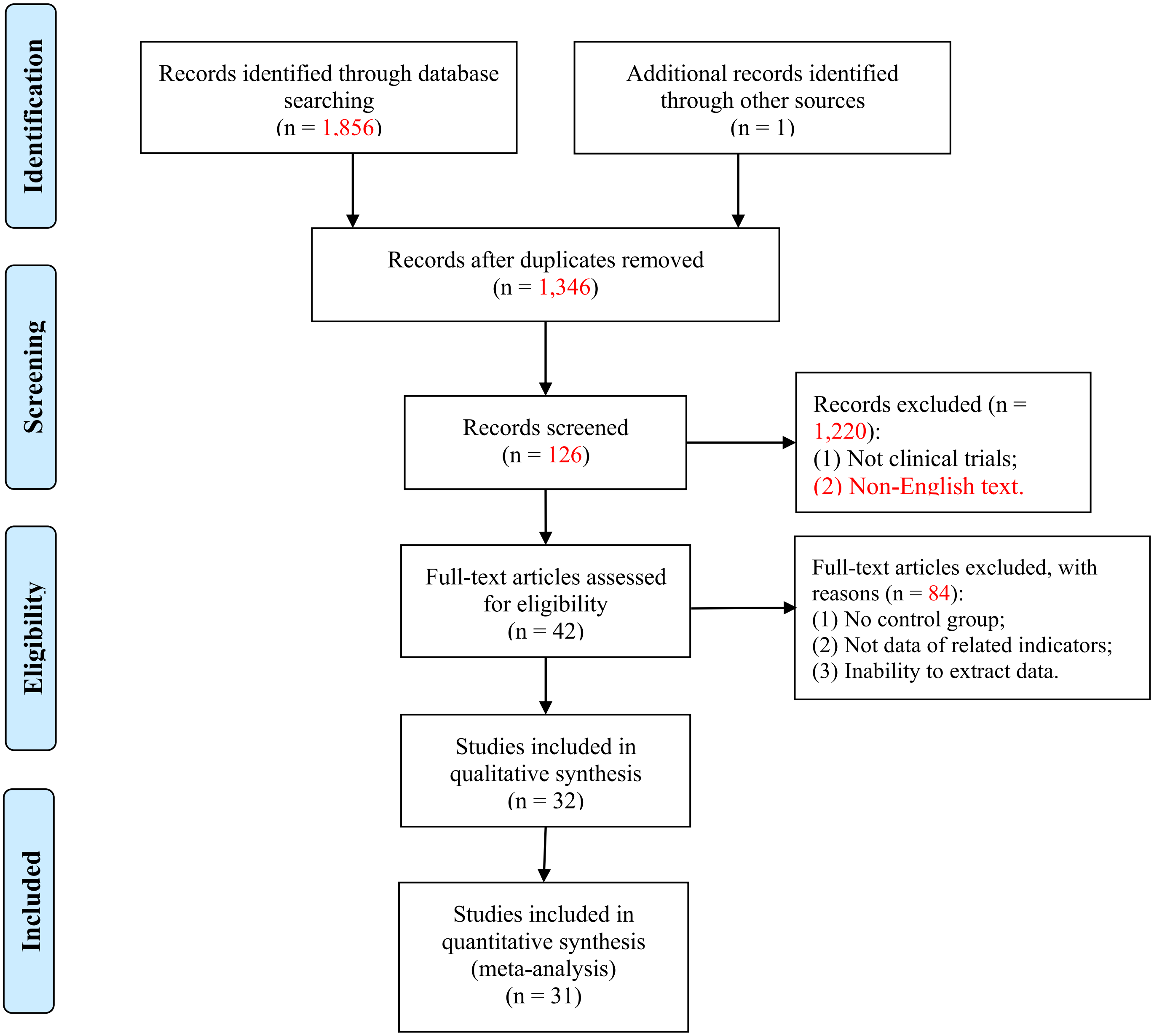

Supplemental Figure 1. The flow diagram of systematic review and meta-analysis.
