# Supplementary material for: Comparisons of the effects of different flaxseed products consumption on lipid profiles, inflammatory cytokines and anthropometric indices in patients with dyslipidemia related diseases: systematic review and a dose–response meta-analysis of randomized controlled trials
Source: Nutr Metab (Lond). 2021 Oct 11;18:91. doi: 10.1186/s12986-021-00619-3 (PMC8504108; doi:10.1186/s12986-021-00619-3)
Supplement: Supplementary file 3 — Additional file 3. Meta-regression investigating the associations between flaxseed-derived product intakes and lipid profiles, inflammatory factors and anthropometric indices. [file 12986_2021_619_MOESM3_ESM.docx]

**Supplemental Table 1.** Meta-regression investigating the associations between flaxseed-derived product intakes and lipid profiles, inflammatory factors and anthropometric indices.

| **Variable** | **Coeﬃcient** | ***SE*** | ***t*** | ***P* _value_** | **95% CI** |
| --- | --- | --- | --- | --- | --- |
| **TC, mg/dL** | | | | | |
| BMI categories | -4.16 | 6.89 | -0.60 | 0.47 | -18.30, 9.99 |
| Design | -4.17 | 9.32 | -0.45 | 0.658 | -23.27, 14.93 |
| Lipid status | 6.17 | 9.06391 | 0.68 | 0.22 | -12.46, 24.80 |
| Type | 21.28 | 7.60 | 2.80 | 0.01* | 5.69, 36.88 |
| Country | 8.49 | 5.31 | 1.60 | 0.12 | -2.38, 19.35 |
| Intervention time | 0.78 | 0.82 | 0.95 | 0.35 | -0.90, 2.46 |
| LDL-C, mg/dL | | | | | |
| BMI categories | -4.20 | 4.69 | -0.90 | 0.58 | -13.82, 5.42 |
| Design | -4.82 | 6.06 | -0.80 | 0.432 | -17.23, 7.58 |
| Lipid status | -11.01 | 4.87 | -2.26 | 0.013* | -21.03, -0.99 |
| Type | 15.53 | 4.13 | 3.76 | 0.001* | 7.05, 24.01 |
| Country | 0.75 | 3.61 | 0.21 | 0.84 | -6.65, 8.15 |
| Intervention time | 5.54 | 3.51 | 1.58 | 0.13 | -1.64, 12.73 |
| HDL-C, mg/dL | | | | | |
| BMI categories | -1.11 | 0.78 | -1.43 | 0.15 | -2.70, 0.48 |
| Design | 0.032 | 1.924 | 0.02 | 0.987 | -3.89, 3.96 |
| Lipid status | 0.67 | 1.38 | 0.49 | 0.76 | -2.15, 3.49 |
| Type | -0.30 | 0.98 | -0.30 | 0.77 | -2.30, 1.71 |
| Country | 1.11 | 0.82 | 1.36 | 0.183 | -0.55, 2.77 |
| Intervention time | 1.75 | 0.80 | 2.20 | 0.036* | 0.13, 3.37 |
| TG, mg/dL | | | | | |
| BMI categories | 17.44 | 15.04 | 1.16 | 0.257 | -13.47, 48.35 |
| Design | 3.57 | 18.45 | 0.19 | 0.848 | -34.29, 41.43 |
| Lipid status | 37.36 | 17.61 | 2.12 | 0.044* | 1.10, 73.62 |
| Type | -8.72 | 17.21 | -0.51 | 0.617 | -44.10, 26.66 |
| Country | -22.18 | 10.35 | -2.14 | 0.041* | -43.42, -0.95 |
| Intervention time | 4.18 | 11.69 | 0.36 | 0.723 | -19.79, 28.16 |
| IL-6, pg/mL | | | | | |
| BMI categories | -0.21 | 0.28 | -0.76 | 0.470 | -0.87, 0.44 |
| Design | 0 .48 | 0.13 | 3.70 | 0.006* | 0.18, 0.79 |
| Lipid status | 0.08 | 0.89 | 0.09 | 0.929 | -2.08, 2.25 |
| Type | 0.23 | 0.30 | 0.77 | 0.465 | -0.46, 0.91 |
| Country | -0.21 | 0.85 | -0.25 | 0.811 | -2.16, 1.74 |
| Intervention time | 0.09 | 0.27 | 0.34 | 0.741 | -0.52, 0.71 |
| hs-CRP, mg/L | | | | | |
| BMI categories | -0.46 | 1.00 | -0.45 | 0.673 | -3.23, 2.32 |
| Design | -0.67 | 0.76 | -0.88 | 0.418 | -2.64, 1.29 |
| Lipid status | -1.4 | 0.61 | -2.30 | 0.105 | -3.34, 0.54 |
| Type | -1.09 | 0.92 | -1.18 | 0.293 | -3.46, 1.29 |
| Country | -1.46 | 0.79 | -1.83 | 0.126 | -3.50, 0.58 |
| Intervention time | 1.28 | 0.64 | 1.99 | 0.104 | -0.37, 2.93 |
| CRP, mg/L | | | | | |
| BMI categories | 0.47 | 1.02 | 0.46 | 0.659 | -1.88, 2.81 |
| Design | 0.42 | 1.42 | 0.29 | 0.775 | -2.80, 3.63 |
| Lipid status | -0.97 | 1.76 | -0.55 | 0.602 | -5.26, 3.33 |
| Type | -0.83 | 0.85 | -0.98 | 0.353 | -2.76, 1.09 |
| Country | -2.09 | 0.63 | -3.34 | 0.009* | -3.51, -0.67 |
| Intervention time | 1.64 | 0.82 | 2.01 | 0.075 | -0.20, 3.49 |
| TNF-α, pg/mL | | | | | |
| BMI categories | -0.61 | 0.54 | -1.14 | 0.338 | -2.33, 1.10 |
| Design | 0.18 | 0.68 | 0.27 | 0.800 | -1.71, 2.08 |
| Lipid status | 0.96 | 0.60 | 1.61 | 0.206 | -0.94, 2.86 |
| Type | -0.288 | 0.23 | -1.26 | 0.265 | -0.88, 0.30 |
| Country | -1.12 | 0.48 | -2.34 | 0.080 | -2.46, 0.21 |
| Intervention time | 0.41 | 0.62 | 0.67 | 0.540 | -1.30, 2.12 |
| BMI, kg/m^2^ | | | | | |
| BMI categories | -0.46 | 0.49 | -0.93 | 0.366 | -1.50, 0.59 |
| Design | -0.19 | 1.35 | -0.14 | 0.892 | -3.06, 2.69 |
| Lipid status | 0.80 | 0.44 | 4.08 | 0.001* | 0.85, 2.75 |
| Type | 0.18 | 0.48 | 0.38 | 0.706 | -0.84, 1.21 |
| Country | -0.947 | 0.438 | -2.16 | 0.047* | -1.88, -0.01 |
| Intervention time | -0.15 | 0.54 | -0.28 | 0.781 | -1.29, 1.00 |
| Weight, kg | | | | | |
| BMI categories | -0.61 | 0.50 | -1.23 | 0.249 | -1.74, 0.51 |
| Design | -0.50 | 3.40 | -0.15 | 0.885 | -8.07, 7.06 |
| Lipid status | -1.6 | 9.86 | -0.16 | 0.875 | -24.33, 21.13 |
| Type | 0.40 | 0.34 | 1.17 | 0.270 | -0.36, 1.15 |
| Country | -0.36 | 0.53 | -0.68 | 0.513 | -1.53, 0.82 |
| Intervention time | -0.04 | 0.33 | -0.13 | 0.896 | -0.77, 0.68 |
| WC, cm | | | | | |
| BMI categories | 1.48 | 1.55 | 0.95 | 0.369 | -2.10, 5.05 |
| Design | -0.61 | 0.62 | -0.99 | 0.347 | -1.99, 0.77 |
| Lipid status | -0.75 | 1.78 | -0.42 | 0.687 | -4.97, 3.47 |
| Type | 1.59 | 1.09 | 1.46 | 0.178 | -0.87, 4.05 |
| Country | -2.81 | 1.22 | -2.30 | 0.047* | -5.56, -0.05 |
| Intervention time | -1.20 | 1.36 | -0.88 | 0.400 | -4.28, 1.88 |

95% CI, 95% confidence interval.

TC, total cholesterol; TG, triglyceride; LDL-C, low-density lipoprotein cholesterol; HDL-C, high-density lipoprotein cholesterol; apo A, apolipoprotein A; apo B, apolipoprotein B; BMI, body mass index; WC, waist circumference.

* with significant difference (*P* < 0.05).
