# Supplementary material for: Comparisons of the effects of different flaxseed products consumption on lipid profiles, inflammatory cytokines and anthropometric indices in patients with dyslipidemia related diseases: systematic review and a dose–response meta-analysis of randomized controlled trials
Source: Nutr Metab (Lond). 2021 Oct 11;18:91. doi: 10.1186/s12986-021-00619-3 (PMC8504108; doi:10.1186/s12986-021-00619-3)
Supplement: Supplementary file 4 — Additional file 4. Subgroup analysis on the effects of flaxseed powder and flaxseed oil on lipid profiles, inflammatory cytokines and anthropometric indices in patients with dyslipidemia related diseases. [file 12986_2021_619_MOESM4_ESM.docx]

**tSupplemental Table 2.** Subgroup analysis on the effects of flaxseed powder and flaxseed oil on lipid profiles, inflammatory cytokines and anthropometric indices in patients with dyslipidemia related diseases

| **Whole flaxseed** | | | | | | **Flaxseed oil** | | | | |
| --- | --- | --- | --- | --- | --- | --- | --- | --- | --- | --- |
| **Subgroups** | **No.** | **Net change (95% CI)** | ***P _value_*** | **I^2^ (%)** | ***P _interaction_*** | **No.** | **Net change (95% CI)** | ***P _value_*** | **I^2^ (%)** | ***P _interaction_*** |
| **Lipid Profiles** | | | | | | | | | | |
| **TC [n=17, -11.85 (-20.12, -3.57), I^2^=97.4%, P= 0.005]** | | | | | | **TC [n=9, 3.35 (-2.89, 9.59), I^2^=29.3%, P=0.293]** | | | | |
| **BMI (kg/m^2^)** |  | | | | |  | | | | |
| <25 and ≥ 25 | 5 | -2.26 (-10.75, 6.24) | 0.602 | 0.0 | 0.00* | 1 | 5.80 (-9.12, 20.72) | 0.446 | ---- | 0.782 |
| ≥25 | 6 | -19.99 (-35.54, -4.43) | 0.012* | 99.1 |  | 7 | 1.74 (-5.98, 9.46) | 0.658 | 44.6 |  |
| ≥30 | 6 | -13.41(-16.41, -10.42) | 0.000* | 2.2 |  | 1 | 6.96 (-8.10, 22.02) | 0.365 | ---- |  |
| **Gender** |  | | | | |  | | | | |
| Mixed | 15 | -12.31 (-21.09, -3.53) | 0.006* | 97.7 | 0.001* | 8 | -0.20 (-7.37, 7.00) | 0.958 | 0.0 | 0.403 |
| Men | 1 | -12.00 (-34.49, 10.49) | 0.296 | ---- |  | 3 | 16.24 (-3.01, 35.49) | 0.098 | 55.4 |  |
| Women | 1 | -3.48 (-22.93, 15.97) | 0.726 | ---- |  | ---- | ---- | ---- | ---- |  |
| **Lipid status** |  | | | | |  | | | | |
| Hypercholesterolemia | 4 | -17.54(-22.26, -12.82) | 0.000* | 45.6 | 0.00* | 1 | -5.80 (-22.59, 10.99) | 0.498 | ---- | 0.373 |
| Mixed hyperlipidemia | 8 | -12.98 (-25.45, -0.48) | 0.042* | 98.7 |  | 5 | 8.87 (-0.29, 18.04) | 0.058 | 45.4 |  |
| Hypertriglyceridemia | 2 | -16.33(-25.04, -7.62) | 0.000* | 0.0 |  | 1 | -3.10 (-21.28, 15.08) | 0.738 | ---- |  |
| Non-dyslipidemia | 3 | -0.85 (-11.07, 9.36) | 0.870 | 0.0 |  | 2 | 1.43 (-10.35, 13.20) | 0.812 | 0.0 |  |
| **Country** |  | | | | |  | | | | |
| Westerner | 12 | -13.53(-16.08, -10.99) | <0.001* | 42.9 | 0.36 | 5 | 6.44 (-8.70, 21.57) | 0.405 | 58.0 | 0.667 |
| Asian | 5 | -19.42(-36.94, -1.91) | 0.03* | 99.2 |  | 4 | 2.16 (-6.11, 10.42) | 0.609 | 0.0 |  |
| **Dose (g/d)** |  | | | | |  | | | | |
| ≤ 30 /≤ 10 | 13 | -13.61 (-22.90, -4.32) | 0.004* | 98.0 | 0.26 | 3 | -0.53 (-11.41,10.35) | 0.923 | 0.0 | 0.244 |
| >30/>10 | 4 | -5.39 (-16.22, 5.45) | 0.330 | 0.0 |  | 5 | 8.11 (-0.44, 16.66) | 0.063 | 42.0 |  |
| **Intervention time (weeks)** | | | | | |  | | | | |
| ≤10 | 8 | -14.49 (-22.89, -6.08) | 0.001* | 88.5 | 0.54 | 3 | -0.51 (-10.94, 9.92) | 0.924 | 0.0 | 0.366 |
| >10 | 9 | -10.43 (-20.23, -0.63) | 0.037* | 90.6 |  | 7 | 5.49 (-2.29, 13.28) | 0.167 | 41.5 |  |
| **LDL-C [n=17, -10.51 (-14.96, -6.06), I^2^=85.3%, P=0.000]** | | | | | | **LDL-C [n=9, -0.00 (-0.003, 0.003), I^2^=0.0%, P=** **0.998]** | | | | |
| **BMI (kg/m^2^)** |  | | | | |  | | | | |
| <25 and ≥ 25 | 5 | -4.51 (-12.43, 3.42) | 0.265 | 0.0 | 0.00* | 1 | 7.40 (-4.91, 19.71) | 0.239 | ---- | 0.214 |
| ≥25 | 6 | -14.83 (-22.62, -7.03) | 0.000* | 94.2 |  | 7 | -0.00 (-0.00, 0.00) | 0.998 | 0.0 |  |
| ≥30 | 6 | -11.01 (-13.53, -8.49) | 0.000* | 0.0 |  | 1 | 7.15 (-3.61, 17.91) | 0.193 | ---- |  |
| **Gender** |  | | | | |  | | | | |
| Mixed | 14 | -10.15 (-15.38, -4.92) | 0.000* | 85.6 | 0.00* | 6 | -0.07 (-0.49, 0.35) | 0.738 | 0.0 | 0.998 |
| Men | 1 | -6.00 (-40.11, 28.11) | 0.730 | ---- |  | 3 | 0.00 (-0.003, 0.003) | 1.000 | 0.0 |  |
| Women | 2 | -11.70 (-14.41, -9.00) | 0.000* | 0.0 |  | ---- | ---- | ---- | ---- |  |
| **Lipid status** |  | | | | |  | | | | |
| Hypercholesterolemia | 4 | -18.74(-22.90, -14.57) | 0.000* | 0.0 | 0.00* | 1 | -0.77 (-16.10, 14.56) | 0.922 | ---- | 0.985 |
| Mixed hyperlipidemia | 8 | -9.28 (-15.17, -3.39) | 0.002* | 89.6 |  | 5 | 0.00 (-0.00, 0.00) | 1.000 | 0.0 |  |
| Hypertriglyceridemia | 2 | -8.64 (-16.59, -0.69) | 0.033* | 0.0 |  | 1 | -0.08 (-0.50, 0.34) | 0.709 | ---- |  |
| Non-dyslipidemia | 3 | -1.27 (-11.33, 8.79) | 0.805 | 0.0 |  | 2 | 0.63 (-11.92, 13.17) | 0.922 | 57.6 |  |
| **Country** |  | | | | |  | | | | |
| Westerner | 12 | -11.61 (-16.22, -6.99) | 0.034* | 47.5 | 0.75 | 5 | -0.00 (-0.003, 0.003) | 1.00 | 0 | 0.749 |
| Asian | 5 | -10.21 (-17.40, -3.02) | <0.001* | 90.6 |  | 4 | -0.07 (-0.49, 0.35) | 0.749 | 26.7 |  |
| **Dose (g/d)** |  | | | | |  | | | | |
| ≤ 30 /≤ 10 | 13 | -10.52 (-15.30, -5.73) | <0.001* | 88.8 | 0.98 | 3 | -0.74 (-8.46, 6.98) | 0.851 | 29.7 | 0.987 |
| >30/>10 | 4 | -10.71 (-21.63, 0.22) | 0.06 | 0.0 |  | 5 | -0.00 (-0.003, 0.003) | 0.998 | 0.0 |  |
| **Intervention time (weeks)** | | | | | |  | | | | |
| ≤10 | 8 | -12.33(-14.27, -10.39) | 0.000* | 0.0 | 0.28 | 3 | 3.04 (-5.28, 11.36) | 0.474 | 0.0 | 0.474 |
| >10 | 9 | -8.04 (-15.50, -0.59) | 0.034* | 83.8 |  | 6 | -0.00 (-0.003, 0.003) | 0.998 | 0.0 |  |
| **HDL-C [n=20, -0.62 (-1.64, 0.40), I^2^=69.6%, P=** **0.236]** | | | | | | **HDL-C [n=9, -0.11 (-1.36, 1.13), I^2^=0.0%, P=** **0.858]** | | | | |
| **BMI (kg/m^2^)** |  | | | | |  | | | | |
| <25 and ≥ 25 | 6 | 1.05 (-1.01, 3.10) | 0.318 | 51.1 | 0.00* | 1 | 0.60 (-2.14, 3.34) | 0.667 | ---- | 0.831 |
| ≥25 | 8 | -0.63 (-1.88, 0.62) | 0.320 | 71.6 |  | 7 | -0.42 (-2.21, 1.38) | 0.650 | 0.0 |  |
| ≥30 | 6 | -2.43 (-3.17, -1.69) | 0.000* | 27.0 |  | 1 | -0.12 (-2.37, 2.13) | 0.917 | ---- |  |
| **Gender** |  | | | | |  | | | | |
| Mixed | 16 | -0.55 (-1.51, 0.41) | 0.263 | 46.8 | 0.354 | 6 | 0.01 (-1.42, 1.44) | 0.989 | 0.0 | 0.863 |
| Men | 1 | -2.00 (-6.18, 2.18) | 0.349 | ---- |  | 3 | -0.56 (-3.09, 2.06) | 0.695 | 0.0 |  |
| Women | 3 | 1.05 (-4.58, 6.69) | 0.714 | 90.2 |  | ---- | ---- | ---- | ---- |  |
| **Lipid status** |  | | | | |  | | | | |
| Hypercholesterolemia | 7 | -0.53 (-4.02, 2.96) | 0.766 | 64.0 | 0.162 | 1 | -1.94 (-8.28, 4.40) | 0.549 | ---- | 0.871 |
| Mixed hyperlipidemia | 8 | -0.97 (-2.16, 0.22) | 0.110 | 82.3 |  | 5 | -0.38 (-2.04, 1.29) | 0.658 | 0.0 |  |
| Hypertriglyceridemia | 2 | 0.84 (-1.93, 3.60) | 0.554 | 0.0 |  | 1 | 0.38 (-3.13, 3.89) | 0.832 | ---- |  |
| Non-dyslipidemia | 3 | -2.49 (-6.14, 1.16) | 0.181 | 0.0 |  | 2 | 0.46 (-1.93, 2.84) | 0.708 | 0.0 |  |
| **Country** |  | | | | |  | | | | |
| Westerner | 15 | -1.07 (-2.88, 0.73) | 0.244 | 51.4 | 0.38 | 5 | -0.85 (-3.15, 1.45) | 0.470 | 0.0 | 0.456 |
| Asian | 5 | -0.10 (-1.28, 1.08) | 0.869 | 78.2 |  | 4 | 0.192 (-1.29, 1.68) | 0.799 | 0.0 |  |
| **Dose (g/d)** |  | | | | |  | | | | |
| ≤ 30 /≤ 10 | 16 | -0.61 (-1.71, 0.49) | 0.279 | 75.4 | 0.93 | 3 | 0.24 (-2.06, 2.55) | 0.836 | 0.0 | 0.813 |
| >30/>10 | 4 | -0.75 (-3.74, 2.24) | 0.623 | 0.0 |  | 5 | -0.16 (-1.69, 1.36) | 0.833 | 0.0 |  |
| **Intervention time (weeks)** | | | | | |  | | | | |
| ≤10 | 10 | -1.73 (-3.67, 0.20) | 0.079 | 79.6 | 0.10 | 3 | -0.45 (-2.52, 1.61) | 0.667 | 0.0 | 0.686 |
| >10 | 10 | 0.38 (-1.22, 1.98) | 0.638 | 50.6 |  | 6 | 0.08 (-1.48, 1.65) | 0.919 | 0.0 |  |
| **TG [n=16, -19.77 (-33.61, -5.94), I^2^=96.1%, P=0.005]** | | | | | | **TG [n=9, 4.45 (-16.88, 25.77), I^2^=64.2%, P= 0.683]** | | | | |
| **BMI (kg/m^2^)** |  | | | | |  | | | | |
| <25 and ≥ 25 | 6 | -3.43 (-19.84, 12.98) | 0.682 | 52.7 | 0.00* | 7 | 8.70 (-21.43, 38.83) | ---- | 0.273 | 0.411 |
| ≥25 | 6 | -25.78 (-52.61, 1.04) | 0.060 | 98.4 |  | 1 | 1.00 (-35.29, 37.29) | 70.8 | 0.571 |  |
| ≥30 | 4 | -35.95 (-65.44, -6.46) | 0.017* | 89.0 |  | 1 | -12.00 (-33.46, 9.46) | ---- | 0.957 |  |
| **Gender** |  | | | | |  | | | | |
| Mixed | 12 | -21.89 (-40.41, -3.37) | 0.02* | 97.0 | 0.008* | 6 | -8.17 (-21.35, 5.00) | 0.224 | 0.0 | 0.712 |
| Men | 1 | 35.0 (106.95, 176.95) | 0.629 | ---- |  | 3 | 30.34 (-41.31, 101.99) | 0.407 | 84.4 |  |
| Women | 3 | -13.95 (-19.79, -8.11) | 0.000* | 0.0 |  | ---- | ---- | ---- | ---- |  |
| **Lipid status** |  | | | | |  | | | | |
| Hypercholesterolemia | 5 | -16.88 (-26.77, -6.98) | 0.001* | 0.0 | 0.00* | 1 | -16.83 (-59.32, 25.66) | 0.438 | ---- | 0.073 |
| Mixed hyperlipidemia | 8 | -18.48 (-38.68, 1.71) | 0.073 | 97.8 |  | 5 | 20.84 (-18.37, 60.05) | 0.298 | 73.9 |  |
| Hypertriglyceridemia | 2 | -52.72(-66.07, -39.37) | 0.000* | 0.0 |  | 1 | -12.40 (-64.87, 40.07) | 0.643 | ---- |  |
| Non-dyslipidemia | 1 | 26.58 (-0.57, 53.73) | 0.055 | ---- |  | 2 | -11.47 (-28.26, 5.32) | 0.181 | 0.0 |  |
| **Country** |  | | | | |  | | | | |
| Westerner | 11 | -11.52 (-19.28, -3.76) | 0.004* | 28.1 | 0.09 | 5 | 17.28 (-25.61, 60.16) | 0.430 | 76.7 | 0.25 |
| Asian | 5 | -39.32 (-70.70, -7.93) | 0.014* | 98.9 |  | 4 | -9.51 (-24.15, 5.12) | 0.203 | 0.0 |  |
| **Dose (g/d)** |  | | | | |  | | | | |
| ≤ 30 /≤ 10 | 13 | -23.52 (-38.28, -8.76) | 0.002* | 96.9 | 0.03* | 3 | -8.33 (-23.97, 7.32) | 0.297 | 0.0 | 0.29 |
| >30/>10 | 3 | 10.50 (-16.49, 37.48) | 0.446 | 0.0 |  | 5 | 16.24 (-26.24, 58.71) | 0.454 | 76.0 |  |
| **Intervention time (weeks)** | | | | | |  | | | | |
| ≤10 | 7 | -17.55 (-47.46, 12.37) | 0.250 | 95.4 | 0.89 | 3 | -1.02 (-24.27, 22.22) | 0.931 | 0.0 | 0.66 |
| >10 | 9 | -19.82 (-33.15, -6.48) | 0.004* | 91.1 |  | 6 | 7.94 (-23.84, 39.71) | 0.624 | 76.6 |  |
| **Apo A [n=4, -4.14 (-4.93, -3.36), I^2^=** **26.2%, P= 0.000]** | | | | | | **Apo A [n=3, -3.19 (-10.67, 4.30), I^2^=** **0.0%, P= 0.404]** | | | | |
| **BMI (kg/m^2^)** |  | | | | |  | | | | |
| <25 and ≥ 25 | 1 | -6.00 (-8.57, -3.43) | 0.000* | 0.0 | 0.138 | ---- | ---- | ---- | ---- | ---- |
| ≥25 | 3 | -3.95 (-4.78, -3.13) | 0.000* | ---- |  | 1 | -5.00 (-15.57, 5.57) | 0.354 | ---- | ---- |
| **Lipid status** |  | | | | |  | | | | |
| Hypercholesterolemia | 2 | -0.54 (-7.59, 6.51) | 0.881 | 0.0 | 0.313 | ---- | ---- | ---- | ---- | ---- |
| Mixed hyperlipidemia | 2 | -4.19 (-4.98, -3.40) | 0.000* | 52.4 |  | ---- | ---- | ---- | ---- | ---- |
| Non-dyslipidemia | ---- | ---- | ---- | ---- | ---- | 1 | -5.00 (-15.57, 5.57) | 0.354 | ---- | ---- |
| **Country** |  | | | | |  | | | | |
| Westerner | 3 | -5.36 (-7.78, -2.94) | 0.00* | 32.9 | 0.96 | 1 | -5.00 (-15.57, 5.57) | 0.354 | ---- | 0.634 |
| Asian | 1 | -4.00 (-4.83, -3.17) | 0.00* | ---- |  | 2 | -1.36 (-11.97, 9.24) | 0.801 | 0.0 |  |
| **Dose (g/d)** |  | | | | |  | | | | |
| ≤ 30 /≤ 10 | 3 | -3.95 (-4.78, -3.13) | 0.0* | 0.0 | 0.14 | 1 | -5.00 (-15.57, 5.57) | 0.354 | ---- | 0.634 |
| >30/>10 | 1 | -6.00 (-8.57, -3.43) | 0.0* | ---- |  | 2 | -1.36 (-11.97, 9.24) | 0.801 | 0.0 |  |
| **Intervention time (weeks)** | | | | | |  | | | | |
| ≤10 | 3 | -5.36 (-7.78, -2.94) | 0.000* | 32.9 | 0.96 | ---- | ---- | ---- | ---- | ---- |
| >10 | 1 | -4.00 (-4.83 -3.17) | 0.000* | ---- |  | 3 | -3.19 (-10.67, 4.30) | 0.404 | 0.0 |  |
| **Apo B [n=4, -5.73 (-7.53, -3.93), I^2^=** **58.1%, P=** **0.000]** | | | | | | **Apo B [n=3, 0.74 (-6.03, 7.50), I^2^=** **0.0%, P= 0.831]** | | | | |
| **BMI (kg/m^2^)** |  | | | | |  | | | | |
| <25 and ≥ 25 | 1 | -7.00 (-9.33, -4.67) | 0.000 | ---- | 0.093 | ---- | ---- | ---- | ---- | ---- |
| ≥25 | 3 | -3.86 (-6.69, -1.02) | 0.008* | 54.0 |  | 1 | -3.00 (-13.37, 7.37) | 0.571 | ---- | 0.351 |
| ≥30 | ---- | ---- | ---- | ---- | ---- | 2 | 3.50 (-5.42, 12.43) | 0.442 | 0.0 |  |
| **Lipid status** |  | | | | |  | | | | |
| Hypercholesterolemia | 2 | -6.92 (-11.53, -2.31) | 0.003* | 38.4 | 0.583 | ---- | ---- | ---- | ---- | ---- |
| Mixed hyperlipidemia | 2 | -4.69 (-9.58, 0.19) | 0.060 | 80.9 |  | ---- | ---- | ---- | ---- | ---- |
| Non-dyslipidemia | ---- | ---- | ---- | ---- | ---- | 1 | -3.00 (-13.37, 7.37) | 0.571 | ---- |  |
| **Country** |  | | | | |  | | | | |
| Westerner | 3 | -6.98 (-9.06, -4.90) | 0.00* | 0.0 | 0.02* | 2 | 3.50 (-5.42, 12.43) | 0.442 | 0.0 | 0.351 |
| Asian | 1 | -2.00 (-5.59, 1.59) | 0.275 | ---- |  | 1 | -3.00 (-13.37, 7.37) | 0.571 | 0.0 |  |
| **Dose (g/d)** |  | | | | |  | | | | |
| ≤ 30 /≤ 10 | 3 | -3.86 (-6.69, -1.02) | 0.008* | 54.0 | 0.09 | 1 | -3.00 (-13.37, 7.37) | 0.571 | 0.0 | 0.351 |
| >30/>10 | 1 | -7.00 (-9.33, -4.67) | 0.000* | ---- |  | 2 | 3.50 (-5.42, 12.43) | 0.442 | ---- |  |
| **Intervention time (weeks)** | | | | | |  | | | | |
| ≤10 | 3 | -6.98 (-9.06, -4.90) | 0.00* | 0.0 | 0.02* | ---- | ---- | ---- | ---- | ---- |
| >10 | 1 | -2.00 (-5.59, 1.59) | 0.275 | ---- |  | 3 | 0.74 (-6.03, 7.50) | 0.831 | 0.0 |  |
| **Inflammatory Factors** | | | | | | | | | | |
| **IL-6 [n=7, -0.17 (-0.42, 0.07), I^2^=** **73.4%, P=** **0.169]** | | | | | | **IL-6 [n=3, -0.35 (-0.67, -0.03), I^2^=** **52.0%, P= 0.033]** | | | | |
| **BMI (kg/m^2^)** |  | | | | |  | | | | |
| <25 and ≥ 25 | 2 | 0.29 (-2.79, 3.36) | 0.856 | 69.8 | 0.376 | ---- | ---- | ---- | ---- | ---- |
| ≥25 | 1 | -0.24 (-0.75, 0.27) | 0.360 | ---- |  | 3 | -0.35 (-0.67, -0.03) | 0.033* | 52.0 | ---- |
| ≥30 | 4 | -0.14 (-0.40, 0.13) | 0.313 | 82.6 |  | ---- | ---- | ---- | ---- | ---- |
| **Gender** |  | | | | |  | | | | |
| Mixed | 6 | -0.046 (-0.25, 0.16) | 0.656 | 48.3 | 0.257 | 2 | -0.15 (-0.53, 0.22) | 0.429 | 0.0 | 0.292 |
| Men | ---- | ---- | ---- | ---- |  | 1 | -0.91 (-1.54, -0.28) | 0.005* | ---- |  |
| Women | 1 | -0.43 (-0.66, -0.20) | 0.000* | ---- |  | ---- | ---- | ---- | ---- |  |
| **Lipid status** |  | | | | |  | | | | |
| Hypercholesterolemia | 1 | -0.24 (-0.75, 0.27) | 0.360 | ---- | 0.00* | 1 | -0.16 (-0.55, 0.23) | 0.419 | ---- | 0.124 |
| Mixed hyperlipidemia | 3 | -0.44 (-0.67, -0.21) | 0.000* | 42.1 |  | 1 | -0.91 (-1.54, -0.28) | 0.005* | ---- |  |
| Hypertriglyceridemia | ---- | ---- | ---- | ---- | ---- | 1 | -0.03 (-1.45, 1.39) | 0.967 | ---- |  |
| **Country** |  | | | | |  | | | | |
| Westerner | 7 | -0.17 (-0.42, 0.07) | 0.169 | 73.4 | ---- | 2 | -0.49 (-1.22, 0.24) | 0.186 | 74.8 | ---- |
| Asian | ---- | ---- | ---- | ---- |  | 1 | -0.03 (-1.45, 1.39) | 0.967 | ---- |  |
| **Dose (g/d)** |  | | | | |  | | | | |
| ≤ 30 /≤ 10 | 6 | -0.13 (-0.39, 0.12) | 0.313 | 75.1 | 0.26 | ---- | ---- | ---- | ---- | ---- |
| >30/>10 | 1 | -0.50 (-1.09, 0.09) | 0.094 | ---- |  | 2 | -0.77 (-1.34, -0.19) | 0.009* | 19.2 |  |
| **Intervention time (weeks)** | | | | | |  | | | | |
| ≤10 | 2 | -0.40 (-0.61, -0.19) | 0.000* | 0.0 | 0.02* | 1 | -0.16 (-0.55, 0.23) | 0.419 | ---- | 0.087 |
| >10 | 5 | -0.02 (-0.25, 0.20) | 0.831 | 53.1 |  | 2 | -0.77 (-1.34, -0.19) | 0.009* | 19.2 |  |
| **hs-CRP [n=5, -0.37 (-1.07, 0.32), I^2^=77.6%, P=0.294]** | | | | | | **hs-CRP [n=2, -1.54 (-2.59, -0.49), I^2^=33.1%, P=0.004]** | | | | |
| **BMI (kg/m^2^)** |  | | | | |  | | | | |
| <25 and ≥ 25 | ---- | ---- | ---- | ---- | ---- | 1 | -1.60 (-2.65, -0.55) | 0.003* | ---- | 0.221 |
| ≥25 | 1 | -0.20 (-0.77, 0.37) | 0.489 | ---- | 0.076 | 1 | 5.82 (-6.03, 17.67) | 0.336 | ---- |  |
| ≥30 | 4 | -0.58 (-1.69, 0.54) | 0.310 | 79.6 |  | ---- | ---- | ---- | ---- | ---- |
| **Gender** |  | | | | |  | | | | |
| Mixed | 6 | -0.66 (-1.35, 0.02) | 0.059 | 37.2 | 0.353 | 2 | 1.54 (-2.59, -0.49) | 0.004 | 33.1 | ---- |
| Men | ---- | ---- | ---- | ---- |  | ---- | ---- | ---- | ---- | ---- |
| Women | 1 | 0.340 (0.25, 0.43) | 0.000 | ---- |  | ---- | ---- | ---- | ---- | ---- |
| **Lipid status** |  | | | | |  | | | | |
| Hypercholesterolemia | 1 | -0.20 (-0.77, 0.37) | 0.489 | ---- | 0.00 | ---- | ---- | ---- | ---- | ---- |
| Mixed hyperlipidemia | 1 | 0.34 (0.25, 0.43) | 0.000 | ---- |  | 1 | -1.60 (-2.65, -0.55) | 0.003* | ---- | 0.221 |
| Non-dyslipidemia | ---- | ---- | ---- | ---- | ---- | 1 | 5.82 (-6.03, 17.67) | 0.336 | ---- |  |
| **Country** |  | | | | |  | | | | |
| Westerner | 4 | -0.29 (-0.97, 0.40) | 0.416 | 80.6 | 0.120 | 1 | 5.82 (-6.03, 17.67) | 0.336 | ---- | 0.221 |
| Asian | 1 | -2.30 (-5.59, 0.99) | 0.170 | ---- |  | 1 | -1.60 (-2.65, -0.55) | 0.003* | ---- |  |
| **Dose (g/d)** |  | | | | |  | | | | |
| ≤ 30 /≤ 10 | 4 | -0.29 (-0.97, 0.40) | 0.416 | 80.6 | 0.120 | 2 | -1.54 (-2.59, -0.49) | 0.004* | 33.1 |  |
| >30/>10 | 1 | -2.30 (-5.59, 0.99) | 0.170 | ---- |  | ---- | ---- | ---- | ---- | ---- |
| **Intervention time (weeks)** | | | | | | | | | | |
| ≤10 | 1 | 0.34 (0.25, 0.43) | 0.000 | ---- | 0.000 | 1 | 5.82 (-6.03, 17.67) | 0.336 | ---- | 0.221 |
| >10 | 4 | -0.51 (-0.96, -0.06) | 0.026* | 37.2 |  | 1 | -1.60 (-2.65, -0.55) | 0.003* | ---- |  |
| **CRP [n=7, -1.09 (-2.51, 0.33), I^2^=** **92.9%, P=** **0.133]** | | | | | | **CRP [n=3, -0.19 (-0.49, 0.11), I^2^= 0.0%, P= 0.220]** | | | | |
| **BMI (kg/m^2^)** |  | | | | |  | | | | |
| <25 and ≥ 25 | 3 | 0.12 (0.05, 0.19) | 0.000 | 0.0 | 0.00 | ---- | ---- | ---- | ---- | ---- |
| ≥25 | 1 | -3.40 (-4.23, -2.58) | 0.000 | ---- |  | 2 | -0.17 (-0.67, 0.33) | 0.502 | 0.0 | 0.686 |
| ≥30 | 3 | -1.13 (-1.89, -0.37) | 0.004* | 45.7 |  | 1 | -0.02 (-0.55, 0.513) | 0.941 | ---- |  |
| **Gender** |  | | | | |  | | | | |
| Mixed | 7 | -1.09 (-2.51, 0.33) | 0.133 | 92.9 |  | 2 | -0.12 (-0.53, 0.29) | 0.561 | 0.0 | 0.643 |
| Men | ---- | ---- | ---- | ---- | ---- | 1 | -0.02 (-0.84, 0.80) | 0.962 | ---- |  |
| Women | ---- | ---- | ---- | ---- | ---- | ---- | ---- | ---- | ---- | ---- |
| **Lipid status** |  | | | | |  | | | | |
| Hypercholesterolemia | 1 | 0.12 (0.05, 0.19) | 0.000 | ---- | 0.00 | 1 | -0.26 (-0.89, 0.37) | 0.417 | ---- | 0.897 |
| Mixed hyperlipidemia | 3 | -1.00 (-3.80, 1.80) | 0.485 | 92.8 |  | 1 | -0.02 (-0.84, 0.80) | 0.962 | ---- |  |
| Hypertriglyceridemia | 1 | -1.03 (-1.80, -0.26) | 0.009 | ---- |  | ---- | ---- | ---- | ---- | ---- |
| Non-dyslipidemia | 2 | -5.13 (-12.14, 1.88) | 0.152 | 45.1 |  | ---- | ---- | ---- | ---- | ---- |
| **Country** |  | | | | |  | | | | |
| Westerner | 5 | 0.12 (0.05, 0.19) | 0.001* | 30.9 | 0.00 | 3 | -0.10 (-0.46, 0.26) | 0.588 | 0.0 | ---- |
| Asian | 2 | -2.21 (-4.53, 0.11) | 0.062 | 94.1 |  | ---- | ---- | ---- | ---- |  |
| **Dose (g/d)** |  | | | | |  | | | | |
| ≤ 30 /≤ 10 | 5 | -0.22 (-0.94, 0.50) | 0.548 | 60.6 | 0.00 | ---- | ---- | ---- | ---- | ---- |
| >30/>10 | 2 | -3.44 (-4.26, -2.63) | 0.000* | 44.1 |  | 2 | -0.02 (-0.47, 0.43) | 0.930 | 0.0 |  |
| **Intervention time (weeks)** | | | | | |  | | | | |
| ≤10 | 2 | -3.38 (-4.18, -2.58) | 0.000* | 0.0 | 0.00 | 1 | -0.26 (-0.89, 0.37) | 0.417 | ---- | 0.524 |
| >10 | 5 | -0.22 (-1.05, 0.61) | 0.602 | 68.6 |  | 2 | -0.02 (-0.47, 0.43) | 0.930 | 0.0 |  |
| **TNF-α [n=6, -0.24 (-0.60, 0.13), I^2^=** **55.6%, P=** **0.200]** | | | | | | **TNF-α (n=0)** | | | | |
| **BMI (kg/m^2^)** |  | | | | |  | | | | |
| <25 and ≥ 25 | 2 | -0.99 (-2.07, 0.09) | 0.073 | 0.0 | 0.116 | ---- | ---- | ---- | ---- | ---- |
| ≥25 | 1 | 0.30 (-0.28, 0.88) | 0.311 | ---- |  | ---- | ---- | ---- | ---- | ---- |
| ≥30 | 3 | -0.30 (-0.77, 0.17) | 0.213 | 71.2 |  | ---- | ---- | ---- | ---- | ---- |
| **Gender** |  | | | | |  | | | | |
| Mixed | 4 | -0.66 (-1.24, -0.08) | 0.027* | 35.1 | 0.265 | ---- | ---- | ---- | ---- | ---- |
| Men | 1 | 0.30 (-0.28, 0.88) | 0.311 | ---- |  | ---- | ---- | ---- | ---- | ---- |
| Women | 1 | -0.03 (-0.14, 0.07) | 0.544 | ---- |  | ---- | ---- | ---- | ---- | ---- |
| **Lipid status** |  | | | | |  | | | | |
| Mixed hyperlipidemia | 4 | -0.03 (-0.13, 0.07) | 0.558 | 30.0 | 0.031* | ---- | ---- | ---- | ---- | ---- |
| Hypertriglyceridemia | 1 | -1.16 (-2.02, -0.30) | 0.008* | ---- |  | ---- | ---- | ---- | ---- | ---- |
| **Country** |  | | | | |  | | | | |
| Westerner | 5 | -0.04 (-0.14, 0.06) | 0.456 | 15.9 | 0.011* | ---- | ---- | ---- | ---- | ---- |
| Asian | 1 | -1.16 (-2.017, -0.30) | 0.008* | ---- |  | ---- | ---- | ---- | ---- |  |
| **Dose (g/d)** |  | | | | |  | | | | |
| ≤ 30 /≤ 10 | 5 | -0.32 (-0.84, 0.19) | 0.217 | 63.2 | 0.533 | ---- | ---- | ---- | ---- | ---- |
| >30/>10 | 1 | -0.20 (-0.67, 0.27) | 0.408 | ---- |  | ---- | ---- | ---- | ---- |  |
| **Intervention time (weeks)** | | | | | |  | | | | |
| ≤10 | 1 | -0.03 (-0.14, 0.07) | 0.544 | ---- | 0.203 | ---- | ---- | ---- | ---- | ---- |
| >10 | 5 | -0.43 (-1.01, 0.15) | 0.149 | 58.5 |  | ---- | ---- | ---- | ---- |  |
| **Anthropometric Index** | | | | | | | | | | |
| **Weight [n=8, -0.40 (-0.76, -0.05), I^2^=** **0.0%, P=0.027]** | | | | | | **Weight [n=4, -0.003 (-0.57, 0.560), I^2^=** **0.0%, P=** **0.911]** | | | | |
| **BMI (kg/m^2^)** |  | | | | |  | | | | |
| <25 and ≥ 25 | 3 | -0.02 (-1.07, 1.02) | 0.966 | 0.0 | 0.560 | 1 | 0.00 (-0.72, 0.72) | 1.000 | ---- | 0.957 |
| ≥25 | 3 | -0.47 (-0.85, -0.09) | 0.016* | 0.0 |  | 2 | -0.86 (-7.40, 5.68) | 0.796 | 0.0 |  |
| ≥30 | 2 | 0.83 (-2.45, 4.10) | 0.621 | 0.0 |  | 1 | 0.08 (-0.86, 1.02) | 0.867 | ---- |  |
| **Gender** |  | | | | |  | | | | |
| Mixed | 7 | -0.42 (-0.77, -0.06) | 0.022* | 0.0 | 0.063 | 4 | -0.003 (-0.57, 0.560) | 0.911 | 0.0 | ---- |
| Men | 1 | 1.00 (-2.33, 4.33) | 0.556 | ---- |  | ---- | ---- | ---- | ---- | ---- |
| Women | ---- | ---- | ---- | ---- | ---- | ---- | ---- | ---- | ---- | ---- |
| **Lipid status** |  | | | | |  | | | | |
| Mixed hyperlipidemia | 7 | -0.40 (-0.75, -0.04) | 0.028* | 0.0 |  | 2 | 0.06 (-0.86, 0.99) | 0.894 | 0.0 | 0.982 |
| Hypertriglyceridemia | ---- | ---- | ---- | ---- | ---- | 1 | -2.90 (-38.53, 32.73) | 0.873 | ---- |  |
| Non-dyslipidemia | ---- | ---- | ---- | ---- | ---- | 1 | 0.00 (-0.72, 0.72) | 1.000 | ---- |  |
| **Country** |  | | | | |  | | | | |
| Westerner | 5 | 0.06 (-0.94, 1.05) | 0.913 | 0.0 | 0.336 | 1 | -0.79 (-7.44, 5.86) | 0.816 | ---- | 0.810 |
| Asian | 3 | -0.47 (-0.85, -0.09) | 0.016* | 0.0 |  | 3 | 0.03 (-0.54, 0.60) | 0.921 | 0.0 |  |
| **Dose (g/d)** |  | | | | |  | | | | |
| ≤ 30 /≤ 10 | 5 | -0.45 (-0.83 -0.08) | 0.019* | 0.0 | 0.423 | 2 | -0.01 (-0.72, 0.70) | 0.980 | 0.0 | 0.885 |
| >30/>10 | 3 | -0.01 (-1.03, 1.01) | 0.983 | 0.0 |  | 2 | 0.08 (-0.86, 1.01) | 0.870 | 0.0 |  |
| **Intervention time (weeks)** | | | | | |  | | | | |
| ≤10 | 3 | -0.49 (-1.12, 0.14) | 0.126 | 0.0 | 0.729 | 2 | 0.06 (-0.86, 1.00) | 0.894 | 0.0 | 0.914 |
| >10 | 5 | -0.36 (-0.79, 0.07) | 0.103 | 0.0 |  | 2 | -0.001 (-0.72, 0.71) | 0.997 | 0.0 |  |
| **BMI [n=9, -0.14 (-0.89, 0.61), I^2^= 77.9%, P=0.719]** | | | | | | **BMI [n=8, -0.03 (-0.24, 0.18), I^2^=0.0%, P=0.770]** | | | | |
| **BMI (kg/m^2^)** |  | | | | |  | | | | |
| <25 and ≥ 25 | 2 | 0.77 (-0.16, 1.69) | 0.105 | 0.0 | 0.00* | 1 | 0.00 (-0.28, 0.28) | 1.000 | ---- | 0.250 |
| ≥25 | 3 | -0.41 (-0.65, -0.17) | 0.001* | 24.8 |  | 6 | -0.14 (-0.46, 0.19) | 0.423 | 0.0 |  |
| ≥30 | 4 | -0.53 (-2.49, 1.43) | 0.597 | 80.9 |  | 1 | 1.55 (-0.47, 3.57) | 0.133 | ---- |  |
| **Gender** |  | | | | |  | | | | |
| Mixed | 8 | -0.21 (-1.05, 0.62) | 0.614 | 79.1 | 0.764 | 7 | -0.04 (-0.25, 0.18) | 0.738 | 0.0 | 0.888 |
| Men | 1 | 0.40 (-0.72, 1.52) | 0.482 | ---- |  | 3 | 0.04 (-1.00, 1.04) | 0.942 | 0.0 |  |
| Women | ---- | ---- | ---- | ---- | ---- | ---- | ---- | ---- | ---- | ---- |
| **Lipid status** |  | | | | |  | | | | |
| Mixed hyperlipidemia | 6 | -0.31 (-0.53, -0.08) | 0.007* | 50.5 | 0.000 | 4 | -0.02 (-0.93, 0.90) | 0.971 | 0.0 | 0.332 |
| Hypertriglyceridemia | 1 | -1.95 (-2.56, -1.34) | 0.000* | ---- |  | 1 | -1.00 (-2.85, 0.85) | 0.288 | ---- |  |
| Non-dyslipidemia | 1 | 1.60 (-2.13, 5.33) | 0.401 | ---- |  | 2 | -0.05 (-0.27, 0.18) | 0.688 | 0.0 |  |
| **Country** |  | | | | |  | | | | |
| Westerner | 5 | 0.62 (-0.07, 1.32) | 0.080 | 0.0 | 0.04* | 5 | 0.25 (-0.59, 1.08) | 0.557 | 0.0 | 0.483 |
| Asian | 4 | -0.70 (-1.74, 0.34) | 0.187 | 87.5 |  | 3 | -0.06 (-0.28, 0.16) | 0.599 | 0.0 |  |
| **Dose (g/d)** |  | | | | |  | | | | |
| ≤ 30 /≤ 10 | 5 | -0.40 (-1.38, 0.57) | 0.419 | 86.0 | 0.17 | 3 | -0.05 (-0.27, 0.17) | 0.671 | 0.0 | 0.766 |
| >30/>10 | 4 | 0.47 (-0.31, 1.24) | 0.238 | 0.0 |  | 5 | 0.08 (-0.73, 0.89) | 0.847 | 0.0 |  |
| **Intervention time (weeks)** | | | | | |  | | | | |
| ≤10 | 3 | -0.37 (-0.61, -0.14) | 0.002* | 55.8 | 0.13 | 1 | -0.28 (-2.50, 1.94) | 0.805 | ---- | 0.831 |
| >10 | 6 | -0.21 (-1.73, 1.32) | 0.792 | 80.3 |  | 7 | -0.04 (-0.25, 0.18) | 0.736 | 0.0 |  |
| **WC [n=7, -0.93 (-3.09, 1.23), I^2^=57.8%, P=** **0.398]** | | | | | | **WC [n=5, -0.93 (-3.09, 1.23), I^2^= 57.8%, P=** **0.398]** | | | | |
| **BMI (kg/m^2^)** |  | | | | |  | | | | |
| <25 and ≥ 25 | 2 | 1.50 (-1.10, 4.11) | 0.258 | 0.0 | 0.444 | ---- | ---- | ---- | ---- | ---- |
| ≥25 | 1 | -0.27 (-1.09, 0.55) | 0.521 | ---- |  | 2 | -0.81 (-2.27, 0.65) | 0.276 | 0.0 | 0.170 |
| ≥30 | 4 | -0.19 (-2.57, 2.18) | 0.873 | 41.1 |  | 3 | 0.77 (-4.84, 6.38) | 0.789 | 73.4 |  |
| **Gender** |  | | | | |  | | | | |
| Mixed | 6 | -0.16 (-1.81, 1.50) | 0.854 | 20.8 | 0.786 | 5 | -0.93 (-3.09, 1.23) | 0.398 | 57.8 |  |
| Men | 1 | 1.00 (-1.66, 3.66) | 0.460 | ---- |  | ---- | ---- | ---- | ---- | ---- |
| Women | ---- | ---- | ---- | ---- | ---- | ---- | ---- | ---- | ---- | ---- |
| **Lipid status** |  | | | | |  | | | | |
| Mixed hyperlipidemia | 4 | -0.02 (-0.77, 0.73) | 0.959 | 0.0 | 0.107 | 1 | -3.10 (-4.81, -1.39) | 0.000 | ---- | 0.038 |
| Hypertriglyceridemia | 2 | -6.46 (-12.40, -0.52) | 0.033* | 0.0 |  | 1 | -1.40 (-6.19, 3.39) | 0.566 | ----- |  |
| Non-dyslipidemia | ---- | ---- | ---- | ---- | ---- | 1 | -0.75 (-2.28, 0.78) | 0.337 | ---- |  |
| **Country** |  | | | | |  | | | | |
| Westerner | 4 | 1.25 (-0.59, 3.09) | 0.182 | 0.0 | 0.111 | 2 | 3.27 (-1.32, 7.86) | 0.163 | 4.2 | 0.036 |
| Asian | 3 | -0.38 (-1.20 0.43) | 0.353 | 51.2 |  | 3 | -1.78 (-2.88, -0.67) | 0.002* | 50.7 |  |
| **Dose (g/d)** |  | | | | |  | | | | |
| ≤ 30 /≤ 10 | 5 | -0.22 (-1.00, 0.56) | 0.583 | 36.2 | 0.378 | 1 | -0.75 (-2.28, 0.78) | 0.337 | ---- | 0.177 |
| >30/>10 | 2 | 1.00 (-1.59, 3.59) | 0.450 | 0.0 |  | 4 | -0.32 (-3.98, 3.34) | 0.864 | 60.8 |  |
| **Intervention time (weeks)** | | | | | |  | | | | |
| ≤10 | 1 | 1.00 (-1.66, 3.66) | 0.460 | ---- | 0.309 | 1 | -3.10 (-4.81, -1.39) | 0.000* | ---- | 0.018 |
| >10 | 6 | -0.21 (-0.99, 0.56) | 0.591 | 20.8 |  | 4 | -0.44 (-1.83, 0.95) | 0.538 | 22.3 |  |

95% CI, 95% confidence interval.

TC, total cholesterol; TG, triglyceride; LDL-C, low-density lipoprotein cholesterol; HDL-C, high-density lipoprotein cholesterol; apo A, apolipoprotein A; apo B, apolipoprotein B; hs-CRP, high sensitivity C-reactive protein; BMI, body mass index; WC, waist circumference.

MetSyn, metabolic syndrome; NAFLD, nonalcoholic fatty liver disease.

Whole flaxseed is bounded by 30 g/d (≤ 30 or > 30); flaxseed oil is bounded by 10 g/d (≤ 10 or > 10).

* with significant difference (*P* < 0.05).
