# Supplementary material for: Comparisons of the effects of different flaxseed products consumption on lipid profiles, inflammatory cytokines and anthropometric indices in patients with dyslipidemia related diseases: systematic review and a dose–response meta-analysis of randomized controlled trials
Source: Nutr Metab (Lond). 2021 Oct 11;18:91. doi: 10.1186/s12986-021-00619-3 (PMC8504108; doi:10.1186/s12986-021-00619-3)
Supplement: Supplementary file 5 — Additional file 5. Meta-regression on linear relationship between dose and absolute changes in lipid profiles, inflammatory cytokines and anthropometric indices. [file 12986_2021_619_MOESM5_ESM.docx]

Meta-regression on linear relationship between dose and absolute changes in TC (mg/dL)

Meta-regression on linear relationship between dose and absolute changes in LDL-C (mg/dL)

Meta-regression on linear relationship between dose and absolute changes in HDL-C (mg/dL)

Meta-regression on linear relationship between dose and absolute changes in TG (mg/dL)

Meta-regression on linear relationship between dose and absolute changes in WC (kg)

Meta-regression on linear relationship between dose and absolute changes in BMI (kg/cm^2^)

Meta-regression on linear relationship between dose and absolute changes in WC (cm)

Meta-regression on linear relationship between dose and absolute changes in IL-6 (pg/mL)

Meta-regression on linear relationship between dose and absolute changes in CRP (mg/L)

Meta-regression on linear relationship between dose and absolute changes in TNF-α (pg/mL)
