# Supplementary material for: Comparisons of the effects of different flaxseed products consumption on lipid profiles, inflammatory cytokines and anthropometric indices in patients with dyslipidemia related diseases: systematic review and a dose–response meta-analysis of randomized controlled trials
Source: Nutr Metab (Lond). 2021 Oct 11;18:91. doi: 10.1186/s12986-021-00619-3 (PMC8504108; doi:10.1186/s12986-021-00619-3)
Supplement: Supplementary file 6 — Additional file 6. Curvilinear regression on nonlinear relationship between dose of whole flaxseed powder and absolute changes inlipid profiles, inflammatory cytokines and anthropometric indices. [file 12986_2021_619_MOESM6_ESM.docx]

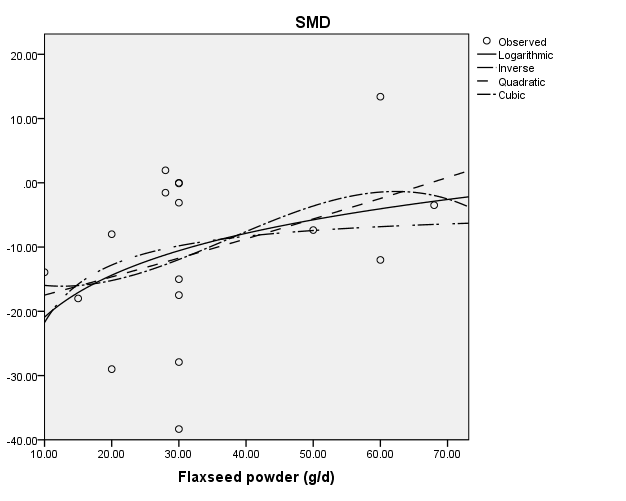


Curvilinear regression on nonlinear relationship between dose of whole flaxseed powder and absolute changes in TC (mg/dL)


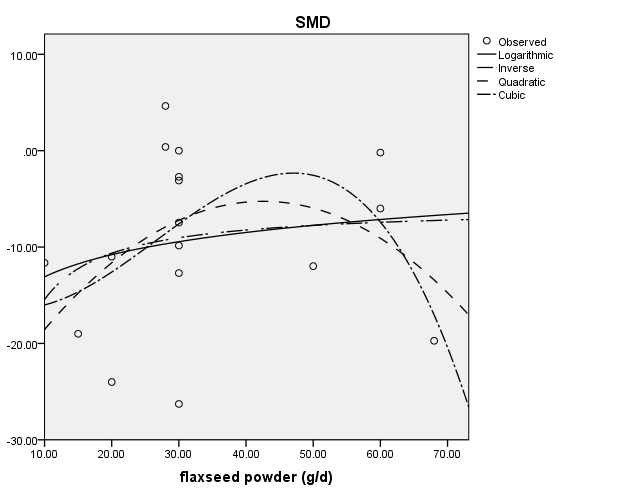


Curvilinear regression on nonlinear relationship between dose of whole flaxseed powder and absolute changes in LDL-C (mg/dL)


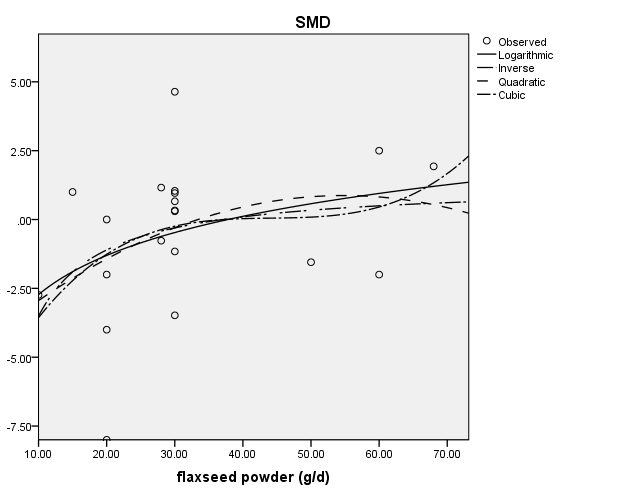


Curvilinear regression on nonlinear relationship between dose of whole flaxseed powder and absolute changes in HDL-C (mg/dL)


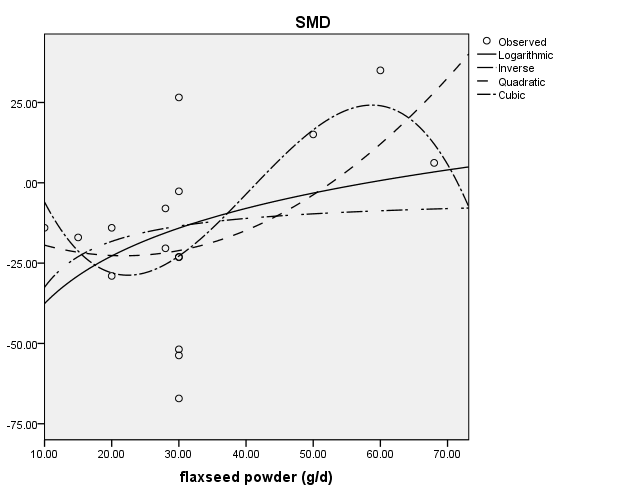


Curvilinear regression on nonlinear relationship between dose of whole flaxseed powder and absolute changes in TG (mg/dL)


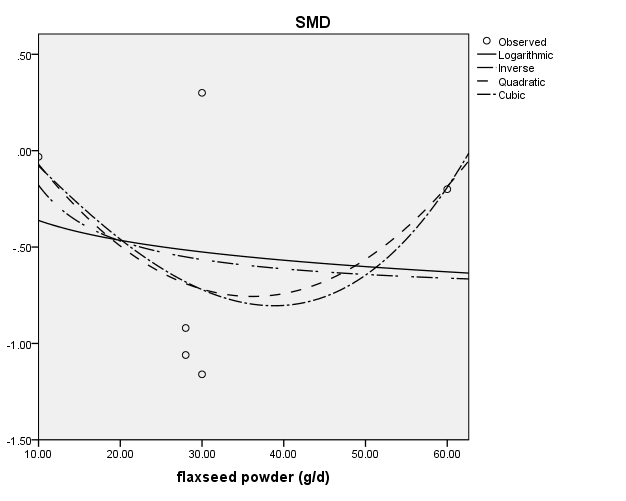


Curvilinear regression on nonlinear relationship between dose of whole flaxseed powder and absolute changes in TNF-α (pg/ml)


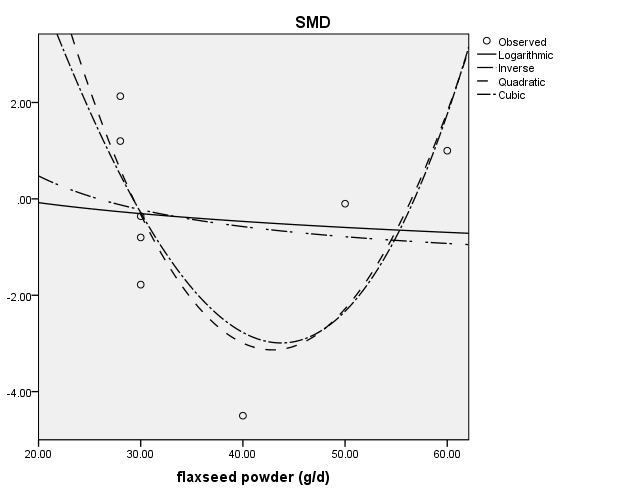


Curvilinear regression on nonlinear relationship between dose of whole flaxseed powder and absolute changes in weight (kg)


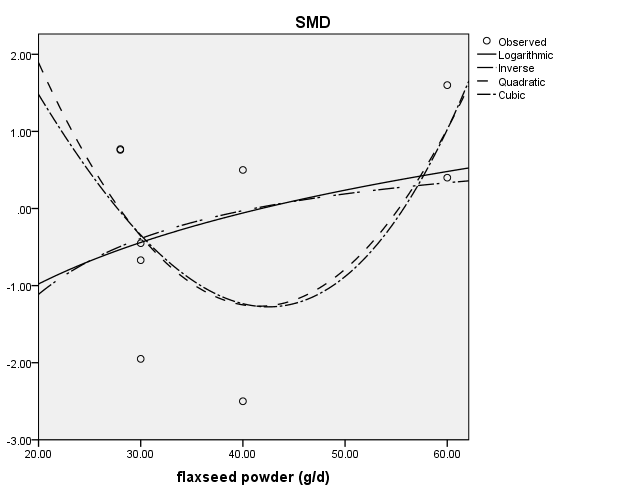


Curvilinear regression on nonlinear relationship between dose of whole flaxseed powder and absolute changes in BMI (kg/cm^2^)


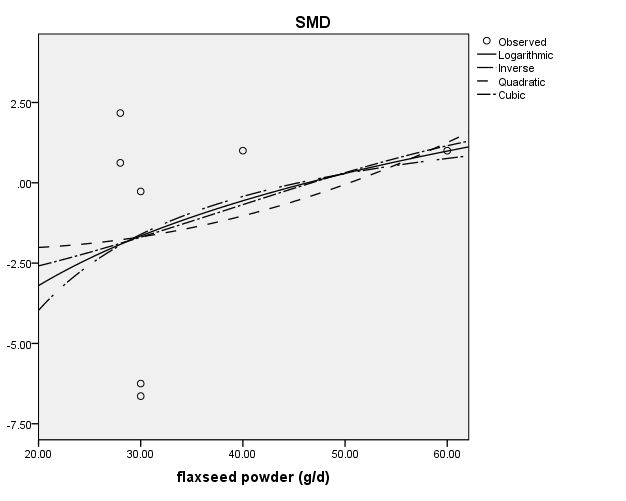


Curvilinear regression on nonlinear relationship between dose of whole flaxseed powder and absolute changes in WC (cm)
